# Quantum Hall phase in graphene engineered by interfacial charge coupling

**DOI:** 10.1038/s41565-022-01248-4

**Published:** 2022-11-21

**Authors:** Yaning Wang, Xiang Gao, Kaining Yang, Pingfan Gu, Xin Lu, Shihao Zhang, Yuchen Gao, Naijie Ren, Baojuan Dong, Yuhang Jiang, Kenji Watanabe, Takashi Taniguchi, Jun Kang, Wenkai Lou, Jinhai Mao, Jianpeng Liu, Yu Ye, Zheng Han, Kai Chang, Jing Zhang, Zhidong Zhang

**Affiliations:** 1 State Key Laboratory of Quantum Optics and Quantum Optics Devices, Institute of Opto-Electronics, Shanxi University, Taiyuan, P. R. China; 2 Shenyang National Laboratory for Materials Science, Institute of Metal Research, Chinese Academy of Sciences, Shenyang, China; 3 Collaborative Innovation Center of Extreme Optics, Shanxi University, Taiyuan, P. R. China; 4 School of Material Science and Engineering, University of Science and Technology of China, Shenyang, China; 5 Liaoning Academy of Materials, Shenyang, China; 6 Collaborative Innovation Center of Quantum Matter, Beijing, China; 7 State Key Lab for Mesoscopic Physics and Frontiers Science Center for Nano-Optoelectronics, School of Physics, Peking University, Beijing, China; 8 School of Physical Science and Technology, ShanghaiTech University, Shanghai, China; 9 ShanghaiTech Laboratory for Topological Physics, ShanghaiTech University, Shanghai, China; 10 College of Materials Science and Optoelectronic Technology, University of Chinese Academy of Sciences, Beijing, China; 11 Research Center for Functional Materials, National Institute for Materials Science, Tsukuba, Japan; 12 International Center for Materials Nanoarchitectonics, National Institute for Materials Science, Tsukuba, Japan; 13 Beijing Computational Science Research Center, Beijing, China; 14 State Key Laboratory for Superlattices and Microstructures, Institute of Semiconductors, Chinese Academy of Sciences, Beijing, China; 15 School of Physical Sciences and CAS Center for Excellence in Topological Quantum Computation, University of Chinese Academy of Sciences, Beijing, China

**Keywords:** Electronic properties and devices, Quantum Hall

## Abstract

The quantum Hall effect can be substantially affected by interfacial coupling between the host two-dimensional electron gases and the substrate, and has been predicted to give rise to exotic topological states. Yet the understanding of the underlying physics and the controllable engineering of this interaction remains challenging. Here we demonstrate the observation of an unusual quantum Hall effect, which differs markedly from that of the known picture, in graphene samples in contact with an antiferromagnetic insulator CrOCl equipped with dual gates. Two distinct quantum Hall phases are developed, with the Landau levels in monolayer graphene remaining intact at the conventional phase, but largely distorted for the interfacial-coupling phase. The latter quantum Hall phase is even present close to the absence of a magnetic field, with the consequential Landau quantization following a parabolic relation between the displacement field and the magnetic field. This characteristic prevails up to 100 K in a wide effective doping range from 0 to 10^13^ cm^−2^.

## Abstract (web summary)

Interfacing graphene with an antiferromagnetic insulator CrOCl enables the observation of strong interfacial coupling in the quantum Hall regime.

## Main

In a number of solid-state systems, the quantum Hall effect (QHE) is found to demonstrate topologically protected dissipation less edge channels with their transversal conductance quantized by *e*^2^/*h*, where *e* and *h* are the elementary charge and the Planck constant, respectively^1–5^. This peculiar behaviour is crucial, for example, in the implementation of quantum-based-resistance standards with an extremely high precision and reproducibility^6^. Among the few known systems that manifest QHE, graphene receives special attention for its distinct band structure and the resulting *N*th Landau level (LL) at the energy of ${\varepsilon }_{{{{\rm{LL}}}}}(N)={{{\rm{sgn}}}}(N){v}_{{\rm{F}}}\sqrt{2e\hslash B| N| }$ under magnetic field *B*, where *v*_F_ is the Fermi velocity^7–9^ and the Landau quantization of graphene in the parameter space of *B* and *n* is defined as the famed Landau fan, with all LLs linearly extrapolated to the charge neutrality point (CNP)^8–10^.

Interfacial coupling is known to affect the QHE in graphene, usually in two different ways: charge impurities that cause a reduced mobility yet a wider quantum Hall (QH) plateaux in some circumstances^6^, and charge transfer that, to some extent, shifts the effective doping^11–16^. Theories predict that the interplay between an antiferromagnetic insulator and graphene can give rise to topological quantum ground states, such as quantum anomalous Hall phases^17–19^. Experimentally, RuCl_3_/graphene is, indeed, spotted with a strong charge transfer, which is sometimes possibly coupled to the magnetism^20^ and sometimes not fully evidenced so^21^.

In this work, we investigate the case of monolayer graphene interfaced with CrOCl, an antiferromagnetic insulator. By examining multiple configurations of graphene encapsulated with hexagonal boron nitride (h-BN) and/or CrOCl, we mapped out the peculiar interfacial coupling between the carbon honeycomb lattice and CrOCl in the parameter space of temperature *T*, total gate doping *n*_tot_, magnetic field *B* and displacement field *D*. At low temperatures, at which the CrOCl bulk was totally insulating, a strong interfacial coupling (SIC) was found in certain gate ranges. At finite magnetic fields, this led to a gate-tunable crossover from fan-like to cascades-like Landau quantization. In the SIC regime, a QHE phase with parabolic dependence between *B* and *D* was obtained in a wide effective doping range from 0 to 10^13^ cm^−2^, with a Landau quantization of a *ν* = ±2 plateau starting from as low as sub-100 mT at 3 K, and remained quantized at ~350 mT at 80 K.

**Fig. 1:**
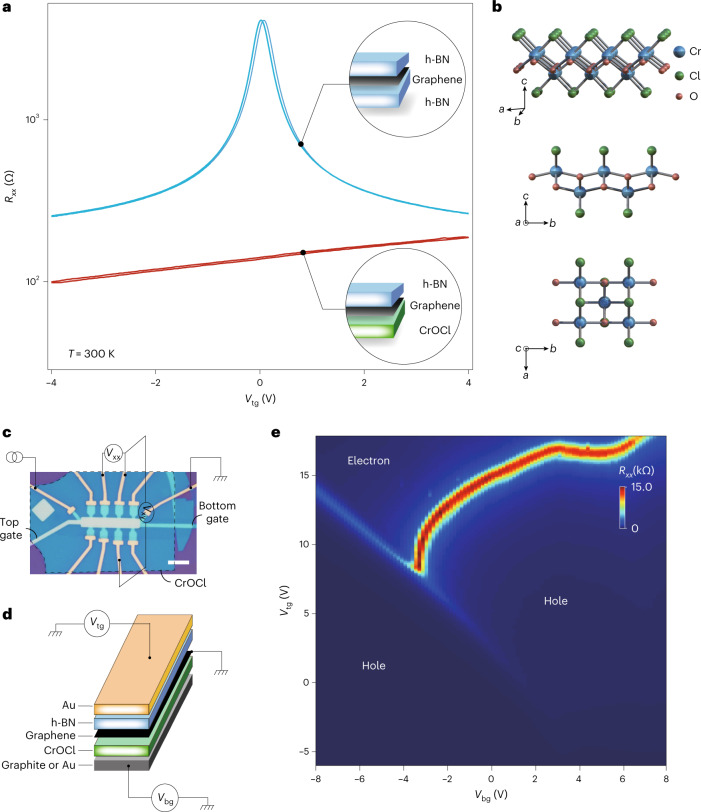
Characterization of CrOCl-supported graphene. **a**, Field effect curves of graphene encapsulated with h-BN and/or CrOCl. Insets: schematic configurations. **b**, Schematics of the crystallographic structure of CrOCl. **c**,**d**, Optical micrograph image of a typical h-BN–graphene–CrOCl sample (**c**), illustrated in **d**. Scale bar, 5 μm. **e**, Colour map of a dual gate scan of the field effect in a typical sample, measured at a temperature of *T* = 3 K and a magnetic field of *B* = 0.

Monolayered graphene, thin CrOCl flakes and encapsulating h-BN flakes were exfoliated from high-quality bulk crystals and stacked in ambient conditions using a dry transfer method^22^. The van der Waals heterostructures were then patterned into Hall bars with their electrodes edge contacted. As seen in Fig. 1a, the field-effect curve of h-BN–graphene–CrOCl samples (red curve) differs from the conventional h-BN–graphene–h-BN ones (blue curve), with the resistive Dirac peak disappearing and a degraded gate tunability (the configurations are illustrated in the Fig. 1a insets). Figure 1b shows the crystal structure of CrOCl (ref. ^23^). We first started with single-gated devices and found that an SIC took place and affected the actual doping in graphene, which exhibited a drastic discrepancy with the doping expected from a conventional gate dielectric, as shown in Supplementary Figs. 1–6.

Figure 1c shows the optical image of a typical h-BN–graphene–CrOCl device, with its structure illustrated in Fig. 1d. A dual-gate mapping of the resistance obtained at *T* =3 K is given in Fig. 1e. Three notable regions are seen, each separated by a resistive peak and marked as either hole or electron doping, as determined by measurements at high magnetic fields discussed below. To further elucidate the SIC in the current system, we define the effective displacement field as *D*_eff_ = (*C*_tg_*V*_tg_ − *C*_bg_*V*_bg_)/2*ϵ*_0_ − *D*_0_, and the induced total carrier of the two gates as *n*_tot_ = (*C*_tg_*V*_tg_ + *C*_bg_*V*_bg_)/*e* − *n*_0_, as commonly used in dual-gated graphene devices^24,25^. Here, *C*_tg_ and *C*_bg_ are the top and bottom gate capacitances per area, respectively, and *V*_tg_ and *V*_bg_ are the top and bottom gate voltages, respectively. *n*_0_ and *D*_0_ are the residual doping and residual displacement field, respectively. Notice that the real doping in graphene *n*_graphene_ can be affected by the interfacial states of CrOCl, whose carrier density is defined as *n*_2_ (Supplementary Note 1), and therefore different from *n*_tot_ in the SIC phase, as is discussed later. Examples of dual-gated maps of channel resistance in the *D*_eff_–*n*_tot_ space are given in Supplementary Figs. 7–12.

**Fig. 2:**
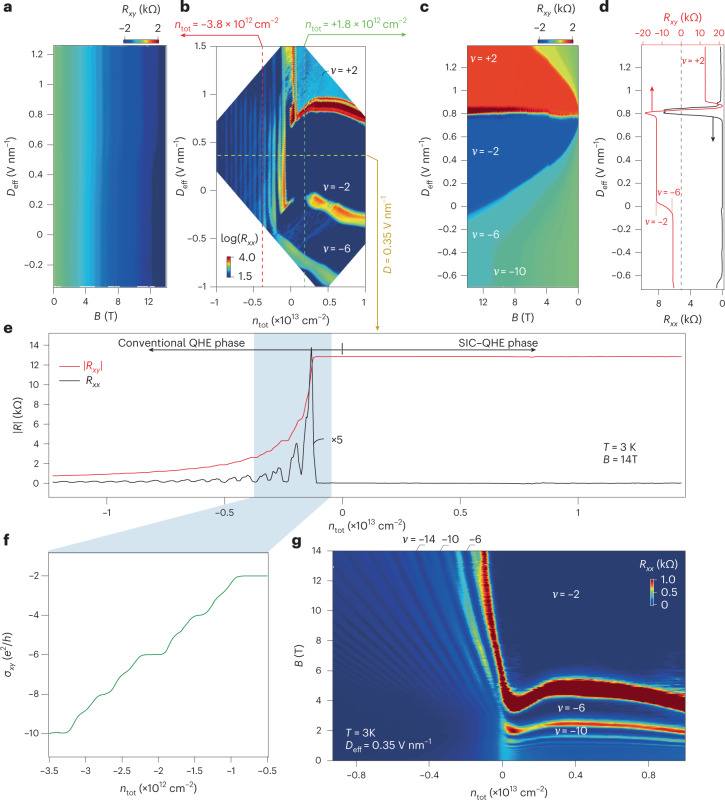
Gate tunable SIC in the QH regime. **a**,**c**, Colour maps of *R*_*xy*_ as a function of *B*, recorded along the red (**a**) and green (**c**) dashed lines in **b**. **b**, *R*_*xx*_ in the parameter space of *D*_eff_ and *n*_tot_, measured at 14 T and 3 K. **d**, Line profiles of *R*_*xx*_ and *R*_*xy*_ at *B* = 12 T in **c**. **e**,**f**, Line profiles along the yellow dashed lines in **b** of *R*_*xx*_ and *R*_*xy*_ at *D*_eff_ = 0.35 V nm^–1^ (**e**), with the zoomed-in *σ*_*xy*_ shown in **f**. **g**, Gate-tunable crossover from fan-like to cascade-like Landau quantization at *D*_eff_ = 0.35 V nm^–1^.

Figure 2a shows a magnetic field scan of *R*_*xy*_ along a fixed carrier density at the hole side with *n*_tot_ ≈ −3.8 × 10^12^ cm^−2^ (red dashed line in Fig. 2b, a mapping of the channel resistance of device-S16 in the *D*_eff_−*n*_tot_ space). Little *D*_eff_ dependence of the filling fraction (that is, LLs) is seen. This is the standard behaviour of monolayer graphene, as there is no *z* dimension and thus the displacement field plays no role in the LLs. Strikingly, as shown in Fig. 2c, a magnetic field scan of transverse resistance *R*_*xy*_ along *n*_tot_ ≈ + 1.8 × 10^12^ cm^−2^ (green dashed line in Fig. 2b) exhibits drastically different patterns as compared with that in Fig. 2a. More details of the carrier types in the dual-gated devices are given in Supplementary Fig. 13. This, as in Fig. 2c, allows one to reach the electron side at *D*_eff_ ≈ 0.8 V nm^–1^, as indicated by the line profiles of both *R*_*xx*_ and *R*_*xy*_ at 12 T in Fig. 2d. In this regime (we call it the SIC–QHE phase), *R*_*xy*_ is quantized in an extremely wide parameter space. For example, at *B* = 14 T, a filling fraction of *ν* = ±2 is found in the effective doping of *n*_tot_ from 0 to 10^13^ cm^−2^ with a displacement field difference δ*D* of over ~2 V nm^–1^, which converts into a very large range of gate voltages.

Figure 2e shows the line profiles of *R*_*xx*_ and *R*_*xy*_ at *D*_eff_ = 0.35 V nm^–1^ (along the yellow dashed line in Fig. 2b) at *B* = 14 T and *T* = 3 K. It is seen that on the hole side (noted as the conventional QHE phase), Landau quantizations are in agreement with those observed in conventional monolayered graphene^8,9^. Full degeneracy lifting with each integer filling fractions from *ν* = –2 to –10 is seen in the zoomed-in window in Fig. 2f. By fitting the hole-side effect curve at a zero magnetic field (Supplementary Fig. 14), the hole carrier mobility was estimated to be about 10^4^ cm^2^ V^−1^ s^−1^. On the positive side of *n*_tot_ in Fig. 2e, the SIC–QHE phase dominated, as the QH plateau of *ν* = –2 extended throughout the whole gate range. By varying the magnetic fields at *D* = 0.35 V nm^−1^, we obtained a colour map in the parameter space of *B* and *n*_tot_, shown in Fig. 2g. It is seen that the SIC led to a change in Landau quantization from the well-known fan-like behaviour to a cascade-like one. To verify *n*_graphene_ as compared with *n*_tot_ in the sample, we extracted *n*_eff_ from the Hall resistance at low fields (that is, *B* < 0.5 T before the quantum oscillation started)—Fig. 2g shows a slope of ~1 with *n*_tot_ at the conventional phase, but a strong departure at a positive *n*_tot_, as shown in Supplementary Fig. 15. Moreover, to have a global picture of the major features described above, the colour maps shown in Fig. 2 were replotted in a three-dimensional presentation, as shown in Supplementary Fig. 16. All these observations were reproducible in multiple samples (Supplementary Figs. 17 and 18), and also confirmed in samples fabricated in a glove box, which ruled out defects in graphene–CrOCl heterostructures (Supplementary Fig. 19).

The central result of this article is the observation of an SIC–QHE phase, in which Landau quantizations seem to be ‘pinned’, such as shown in Fig. 2g. A trivial explanation for this would be that the charge accumulation at the interface of graphene–CrOCl screened the positive gate voltages applied, which leads to a failure of electron injection. However, as shown in Fig. 2c, *D*_eff_ totally shuffles the LLs (hence the *n*_graphene_), which rules out the ‘charge pinning’ picture, as it would then be *D* independent, as in the conventional QHE phase (such as in Fig. 2a). Moreover, the Landau quantization seemed to approach the *B* = 0 limit in the SIC–QHE phase, as shown in Fig. 2c.

**Fig. 3:**
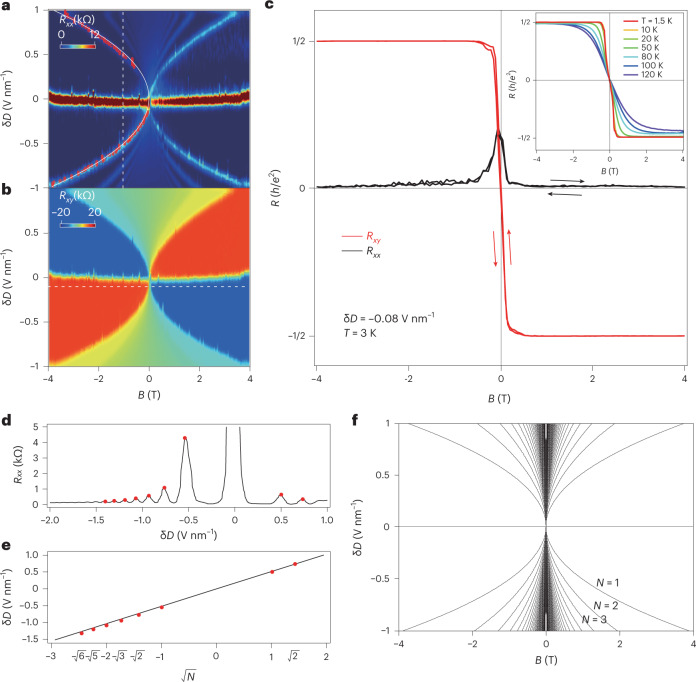
Characteristics of the SIC–QHE phase in graphene–CrOCl heterostructures. **a**,**b**, *R*_*xx*_ (**a**) and *R*_*xy*_ (**b**) of device-S16 plotted in the parameter space of δ*D* and *B*. **c**, Line profiles of *R*_*xx*_ and *R*_*xy*_ at δ*D* = –0.08 V nm^–1^. Inset: temperature dependence of another typical sample (device-S40) at δ*D* = –0.15 V nm^–1^. **d**, Line profile of *R*_*xx*_ in **a** at *B* = –1 T (indicated by the vertical white dashed line). Red dots are resistive peaks picked by each maximum. **e**, Dependence of δ*D* and $\sqrt{N}$. The black solid line is a linear fit. **f**, Parabolic dependence of $\updelta D=\alpha \sqrt{B|N| }$, plotted with *α* = 0.513 and ∣*N*∣ < 200.

To further clarify this perplexing scenario, we carried out a zoomed-in scan of the low magnetic field part of Fig. 2c. We defined the displacement field in which the carrier type switches from holes to electrons as *D*_neutral_, and thus the *D* axis was renormalized as δ*D* = *D* − *D*_neutral_. As shown in Fig. 3a,b (*R*_*xx*_ and *R*_*xy*_, respectively), wide Landau plateaux are seen. The quantized regions touch the *B* = 0 T line, and a tiny width still exists in the vicinity of a zero magnetic field. The *D*–*B* relation of LLs observed here is distinct from those found in other multilayered graphene systems^26–28^. We took the δ*D* = –0.08 V nm^–1^ here (indicated by the white dashed line in Fig. 3b), and plotted both *R*_*xx*_ and *R*_*xy*_ (Fig. 3c). The curves show a well-quantized plateaux of *R*_xy_= ± 0.5*h*/*e*^2^ starting from *B* as low as sub-100 mT, at which *R*_*xx*_ shows near-zero reminiscent values at each plateau. Although a quantum anomalous Hall effect (QAHE) or Chern insulator is claimed in graphene systems^29–32^, our device with a h-BN–monolayer-graphene–CrOCl heterostructure seems to be topologically trivial when a magnetic field is completely absent, and the observed *R*_*xy*_ quantization at a very low *B* is still in the regime of QH states, as the quantization of *ν* = ± 2 is inherited from the Dirac electrons, and no magnetic hysteresis (that is, the coercive field) is seen in the trace–retrace loop of a magnetic scan in our system (indicated by arrows in Fig. 3c, and see Supplementary Fig. 20). More discussion can be seen in Supplementary Note 2. A trivial effect of gate leakage was ruled out, and multiple samples were tested to a maximum temperature before gate leakage took place, shown in Supplementary Figs. 21–23. Notably, this robust SIC–QHE phase in the graphene/CrOCl heterostructure prevails at much higher temperatures (Fig. 3c inset).

By extracting a line profile of *R*_*xx*_ in Fig. 3a at *B* = –1 T (indicated by the vertical white dashed line), resistive peaks were found at each LL, as indicated by the red dots in Fig. 3d. It was found that the δ*D* values at each resistive peak were in linear dependence with $\sqrt{N}$, with *N* the *N*th LL, shown in Fig. 3e. This is a typical Landau quantization energy dependence in conventional monolayered graphene. Indeed, the δ*D*–*B* relation can be fitted using a parabolic curve as $\updelta D=\alpha \sqrt{B| N| }$. The peaks of *R*_xx_ of the first LL in Fig. 3a (red circles) were fitted with a white solid parabolic curve, with *α* = 0.513. The first 200 LLs were then plotted (Fig. 3f), and well simulated the experimental δ*D* data. This indicates that δ*D* linearly tunes the chemical potential of the LLs of graphene, which stimulated us to propose a possible mechanism to explain the SIC–QHE as outlined in the following section. Interestingly, the observed SIC–QHE phase seems to have no connection to the antiferromagnetic nature of CrOCl, as its Néel temperature is ~13 K (ref. ^23^), much lower than the upper bound temperature for the SIC–QHE phase. In addition, we noticed that a sister compound of CrOCl, FeOCl, is far less stable, and could not be used to check the universality of the findings in this work (Supplementary Fig. 24).

**Fig. 4:**
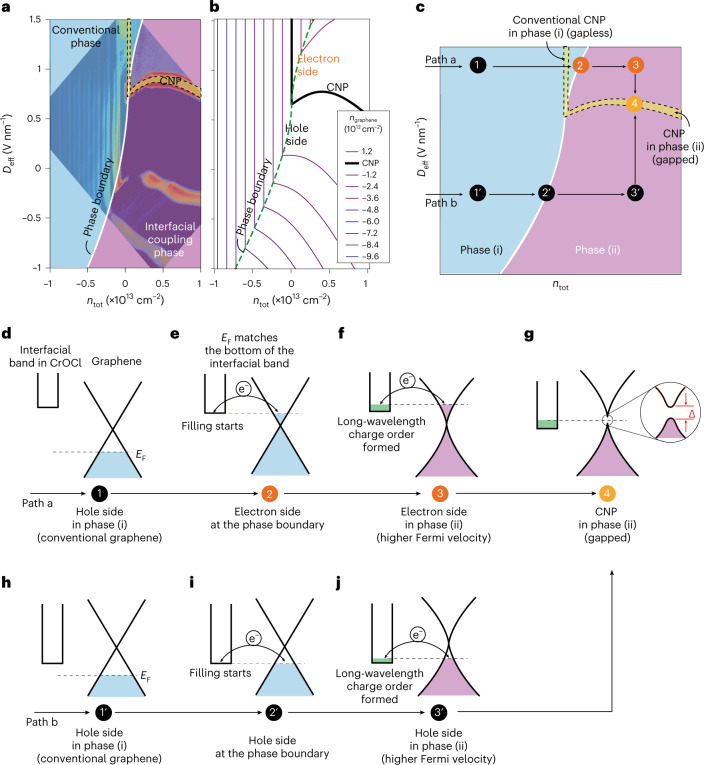
QH phase diagram in the *D*_eff_–*n*_tot_ space and the transition processes between phases. **a**,**b**, Experimental (**a**) and calculated (**b**) phase diagram in the *D*_eff_–*n*_tot_ space, with the phase boundary highlighted. Iso-doping lines with the calculated *n*_graphene_ are indicated by solid lines in **b**. **c**, Schematic of a typical phase diagram with two paths of transition processes noted by arrows. **d**–**j**, Schematics of the band diagrams (steps (1)–(4)) for path a (**d**–**g**, respectively) and those (steps (1′)–(3′) for path b (**h**–**j**, respectively) illustrated in **c**.

We then replotted (Fig. 4a) the *R*_*xx*_ of device-S16 in the *D*_eff_–*n*_tot_ space at 14 T with false colour that separates the boundary between the conventional and SIC phases, and the LLs naturally denote iso-doping lines, as defined by *ν* = *hn*_graphene_/*eB*. Two key features are seen in Fig. 4a. First, the CNP is bent as the system enters from conventional phase into the SIC phase. Second, each spacing between the iso-doping lines increases as the system enters deeper into the SIC phase. We propose an electrostatic model in Supplementary Note 1. An interfacial band with considerable charge density of *n*_2_ is introduced at the surface of CrOCl with a distance *d*_2_ below the graphene layer, and the top and bottom gates are located at distances *d*_1_ and *d*_3_, respectively, as illustrated in Supplementary Fig. 25a,b. By evaluating the model, we found that these two major features can be well reproduced, as shown in the phase diagram in Fig. 4b. Nevertheless, in the simplified model we had to introduce two assumptions—a band structure reconstruction with an enhanced Fermi velocity once the Fermi level of graphene becomes aligned with the interfacial band in CrOCl, and also that the interfacial band exhibits no contribution to transport, as discussed in Supplementary Figs. 25–28.

We further performed density functional theory calculations (Supplementary Figs. 29–31 in Supplementary Note 2). It is seen that, in the bilayered CrOCl model and at certain vertical electric fields, the interfacial band from the top layer of CrOCl (Supplementary Figs. 32–34) starts to overlap with the Fermi level of graphene. The charge transfer from graphene to the interfacial band is thus allowed via tunnelling. Our calculations suggest that a long-wavelength localized charge order (a Wigner crystal, in this case, as the dimensionless Wigner–Seitz radii are estimated to exceed the critical value of 31 for two-dimensional electrons,^33^ shown in Supplementary Table 1) is likely to form in the interfacial band of the Cr 3*d* orbitals in the top layer of CrOCl. This self-consistently explains that, once filled with electrons, the interfacial band may undergo a Wigner instability and does not contribute to transport, but provides a superlattice of Coulomb potential for the graphene resting on top. When systematically considering the interplay between generic long-range Coulomb superlattice potentials in a number of materials coupled with graphene, our separate theoretical work suggests that such e−e interaction in graphene indeed enhances the Fermi velocity dramatically and in the meantime opens a gap at the CNP^34^. It came to our notice that similar phenomena were also recently seen, such as in graphene–CrI_3_ system^35^.

Based on the above analysis, we plotted a schematic phase diagram (Fig. 4c), in which the conventional and SIC phases were denoted as phase (i) and phase (ii) for simplicity. Two different paths are used to illustrate the doping processes in our system. In path a, graphene starts in a hole-doped state (state (1) in Fig. 4d). It crosses the CNP, becomes electron doped and approaches the phase boundary at which the Fermi level of graphene touches the lowest energy of the interfacial band in CrOCl (state (2) in Fig. 4e), which thus triggers the electron-filling event in the interfacial band and forms a charge order. The latter exerts a long-wavelength Coulomb superlattice potential to the Dirac electrons in graphene. Consequently, the Fermi velocity is notably enhanced (sharpening of the Dirac cone in the illustration) driven by e–e interactions in graphene (state (3) in Fig. 4f). Furthermore, when *D*_eff_ is decreased from state (3) to state (4), the Fermi level in graphene reaches its CNP, at which an interaction-driven gap is seen (as supported by the extraction of thermal activation gap; Supplementary Fig. 35). On a further decrease in *D*_eff_, the system becomes hole-doped again. A similar process can be interpreted for path b (Fig. 4h–j).

**Fig. 5:**
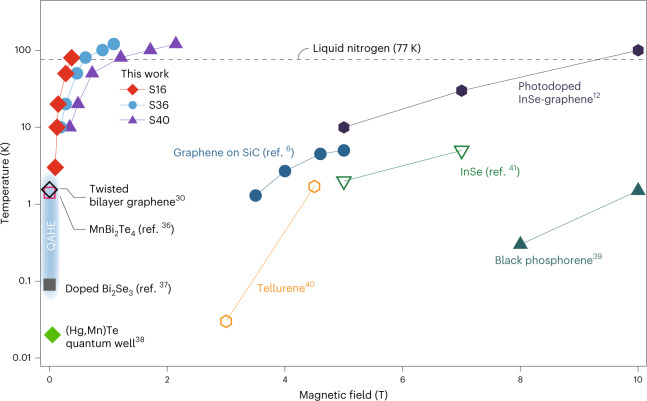
Perspectives for the SIC–QHE phase. The diagram summarizes magnetic fields (below 10 T) and temperatures that realize quantized Hall conductance in several typical systems reported recently. Data from three samples (devices S16, S36 and S40) in this work are included.

Experimentally, by fitting the Shubnikov–de Haas oscillations from various temperatures at dopings in phase (i) and phase (ii) (Supplementary Fig. 36), the cyclotron mass m* in phase (i) was estimated to be comparable to that in ‘ordinary’ monolayer graphene, but 3–5 times larger than that in phase (ii). It hence yields a Fermi velocity a few times larger than that of graphene in phase (ii), in agreement with the conjectures in our theoretical model. Thus, in this regime the cyclotron gap of the first LL, $\Delta ={v}_{{\rm{F}}}\sqrt{2\hslash eB}$, is in the order of about 50 meV at 0.1 T, which qualitatively explains the quantization at a very low *B*. We emphasize that further probes, such as infrared transmission, would help to directly verify the cyclotron gap estimated in the current system in this regard. A robust QH state with ultralow magnetic fields at relaxed experimental conditions can be crucial for future constructions of topological superconductivity as well as quantum-information processing, which has long thought to be only possible in QAHE systems. The above results unambiguously show that the interfacial charge coupling, in terms of engineering the quantum electronic states, is a powerful technique that we may have overlooked thus far. For comparison, Fig. 5 summarizes the magnetic fields and temperatures required to realize quantized Hall conductance in typical different QHEs or QAHE systems reported recently^6,12,30,36–41^.

In conclusion, we have demonstrated a hybrid system of graphene–CrOCl, in which an exotic QHE phase was observed thanks to the peculiar gate tunable interfacial coupling. At finite magnetic fields and constant *D*_eff_, a crossover from fan-like to cascade-like Landau quantization is seen. Also, in the *D*–*B* space, unlike in conventional *D*-independent ones, the LLs in the SIC–QHE phase exhibits a parabolic dependence between *B* and *D*_eff_ in a wide effective doping range from 0 to 10^13^ cm^−2^, with a Landau quantization of a *ν* = ±2 plateau starting from as low as sub-100 mT below 10 K, and remains quantized at ~350 mT at liquid nitrogen temperature. Our theoretical analysis self-consistently attributes the physical origin of this observed phenomenon to the formation of a long-wavelength charge order in the interfacial states in CrOCl and a subsequent band reconstruction in graphene. Our findings seem to open a new door to engineering the QH phase, and may shed light on the future manipulation of quantum electronic states via interfacial charge coupling, such as to construct novel topological superconductors, and to build quantum metrology standards.

## Methods

### Sample fabrication and characterization

The CrOCl–graphene–h-BN heterostructures were fabricated in ambient conditions using a dry-transfer method, with the flakes exfoliated from high-quality bulk crystals. CrOCl layers were etch patterned using an ion milling with Ar plasma, and dual gated samples were fabricated using standard electron-beam lithography. A Bruker Dimension Icon atomic force microscope was used to measure thickness, morphology and surface potential. The electrical performances of the devices were measured using a BlueFors LD250 at millikelvin temperature, a Quantum Design PPMS system was used for temperature (3–300 K) and magnetic field (±14 T) scans and a probe station (Cascade Microtech Inc. EPS150) for room-temperature electrical tests.

### Density functional theory calculations

The first-principles calculations based on density functional theory were carried out with Vienna ab initio Simulation Package with a projector augmented wave method^42,43^. The plane-wave energy cutoff was set to be 600 eV, and the crystal structure was fully relaxed until the residual forces on the atoms were less than 0.01 eV Å^–1^. The generalized gradient approximation by Perdew, Burke and Ernzerhof was taken as the exchange-correlation potential^44^. As Cr is a transition metal element with localized 3*d* orbitals, the on-site Hubbard *U* = 2.7 eV parameter was used in the calculations. To properly include the effects of vertical electric fields, we focused on bilayer CrOCl because the monolayer is indifferent to electric fields. We considered three magnetic configurations in CrOCl, and the thickness of the vacuum region was set as 40 Å to avoid any artificial interactions. The ‘DFT+D2’ type of van der Waals correction was adopted for the bulk calculations to properly describe the interlayer interactions^45,46^. The so-called fully localized limit of the spin-polarized GGA+U functional was adopted, as suggested by Liechtenstein and co-workers^47–50^. In the calculations of the commensurate supercell of the bilayer-CrOCl–graphene heterostructure, a 2 × 4 supercell for the bilayer CrOCl, and a $3\times 3\sqrt{3}$ supercell for monolayer graphene were adopted.

## Online content

Any methods, additional references, Nature Portfolio reporting summaries, source data, extended data, supplementary information, acknowledgements, peer review information; details of author contributions and competing interests; and statements of data and code availability are available at 10.1038/s41565-022-01248-4.

## Supplementary information

Supplementary Information Supplementary Information Supplementary Figs. 1–36, Table 1 and Notes 1 and 2.

## Data Availability

The data that support the findings of this study are available at Zenodo, 10.5281/zenodo.7046671.

## Code availability

The codes used in theoretical simulations and calculations are available from the corresponding authors upon reasonable request.
