# Supplementary material for: Quantum Hall phase in graphene engineered by interfacial charge coupling
Source: Nat Nanotechnol. 2022 Nov 21;17(12):1272–9. doi: 10.1038/s41565-022-01248-4 (PMC9747608; doi:10.1038/s41565-022-01248-4)
Supplement: Supplementary file 1 — Supplementary Information Supplementary Figs. 1–36, Table 1 and Notes 1 and 2. [file 41565_2022_1248_MOESM1_ESM.pdf]

nature nanotechnology

Article https://doi.org/10.1038/s41565-022-01248-4

Quantum Hall phase in graphene engineered by interfacial charge coupling

In the format provided by the authors and unedited

Outlines

1. Characterization of single gated graphene/CrOCl heterostructures

2. Scanning tunneling microscope morphology of monolayered graphene on few-layered CrOCl

3. Characterization of dual gated graphene/CrOCl heterostructures

4. Reproducibility of the observed exotic phenomenon in multiple samples

5. Hysteresis analysis of the dual gate scans by sweeping the gate voltages along different directions in Device-S13

6. Plotting the dual gated map of channel resistance into the space of D_{eff}-n_{tot}

7. Carrier doping in dual gated h-BN/graphene/CrOCl device in the space of D_{eff}-n_{tot}

8. Hole mobility in a typical dual-gated Au/CrOCl/graphene/h-BN/Au device

9. Line profile of n_{graphene} obtained from Hall measurement as a function of n_{tot}, showing the difference between n_{graphene} and n_{tot}

10. A presentation of the "data cube" in the pace of B, n_{tot}, and D_{eff} of the currently studied system

11. Control samples showing similar data to the one in Fig. 2 in the main text

12. Data obtained in typical sample fabricated in a glove box

13. Zoomed-in map of channel resistance in the δD-B space

14. Ruling out trivial effect of gate leakage and testing multiple samples in the space of δD-B to a maximum temperature before gate leakage takes place

15. Air instability of FeOCl, a sister compound of CrOCl

Supplementary Note 1. The electrostatic model of the h-BN/Graphene/CrOCl

Supplementary Note 2. DFT calculations with the Hartree-Fock methods

16. Thermal gap extracted from the CNP at the SIC-phase

17. Shubnikov-de Haas (SdH) analysis of the h-BN/graphene/CrOCl heterostructures

1. Characterization of single gated graphene/CrOCl heterostructures

It is noticed that, once contacted with graphene, the few-layered CrOCl exhibits weak bulk conductivity at temperatures above ~200 K, as shown in Supplementary Figure 1-2. Therefore, only top gate is allowed at room temperature, as bottom gate leakage currents will occur.

When cooled down, those CrOCl interfaced graphene samples equipped with one single bottom gate showed a restore of bipolar type of field effect curve, as if the sample was back to "normal" graphene (Supplementary Figure 3). However, at finite magnetic fields, Landau fans in them are found to be asymmetric with respect to the presumed charge neutrality point, with the transition from a fan-like diagram in the hole side to a cascades-like diagram in the electron side (Supplementary Figure 4). Low field ($B<0.5$ T) Hall coefficient analysis indicates that hole carrier type dominates in the whole gate range, even though the sample is set to a positive gate voltage (Supplementary Figure 5a-b). This is a strong indication that a peculiar strong interfacial coupling (SIC) is taking place at the interface of CrOCl and graphene.

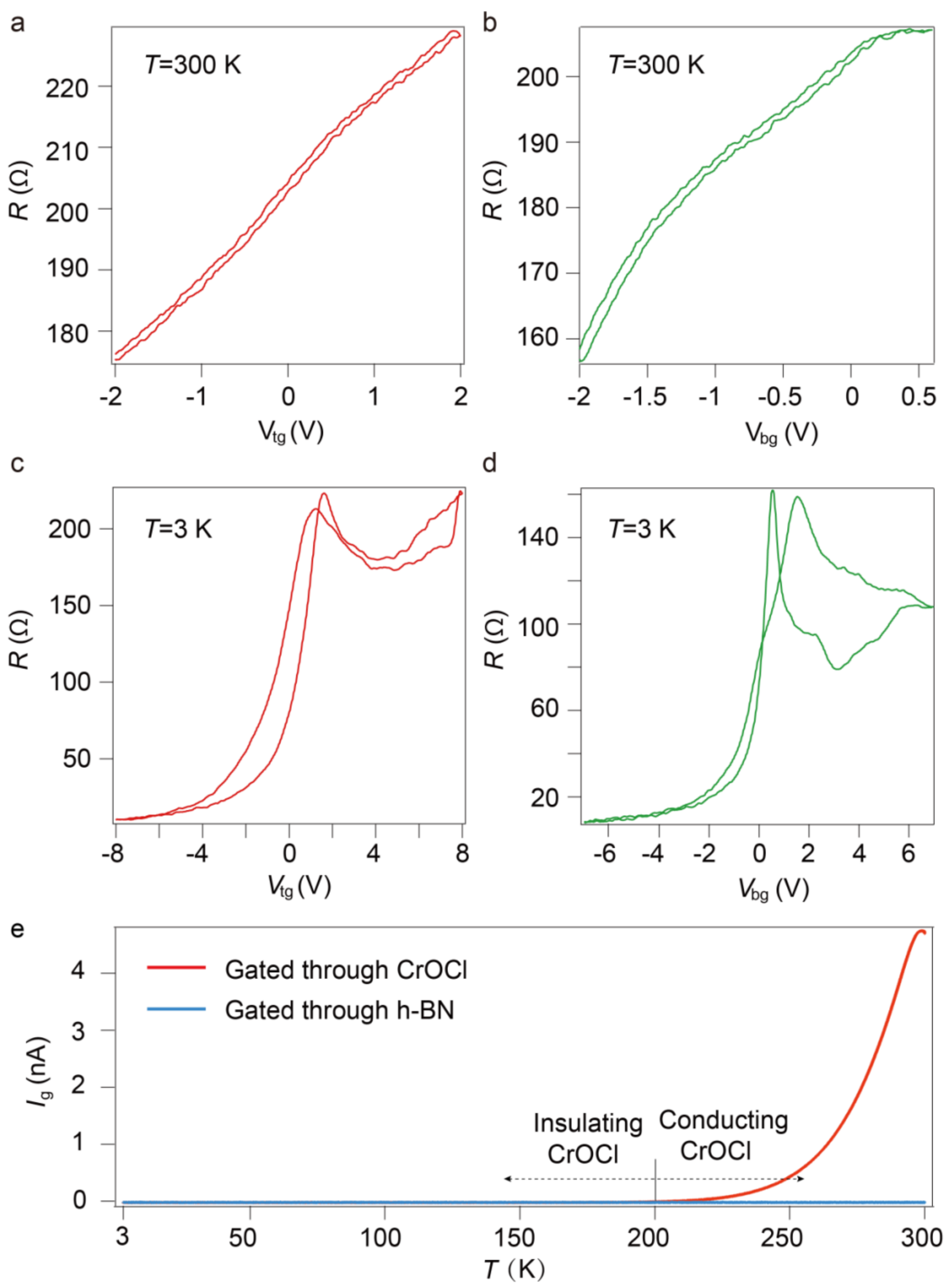

Supplementary Figure 1. Weak bulk conductivity above 200 K in the h-BN/graphene/CrOCl heterostructure.(a) and (b) show transfer curves of the device gated at T=300 K through h-BN, and CrOCl, respectively. The corresponding transfer curves at 3 K are shown in (c) and (d). (e) Leakage current from gate electrodes recorded through different dielectrics. During the test, gate voltages of 0.5 V were applied. It is seen that graphene-contacted few-layered CrOCl exhibits bulk conductivity above 200 K, while h-BN is a reference for the insulating state.

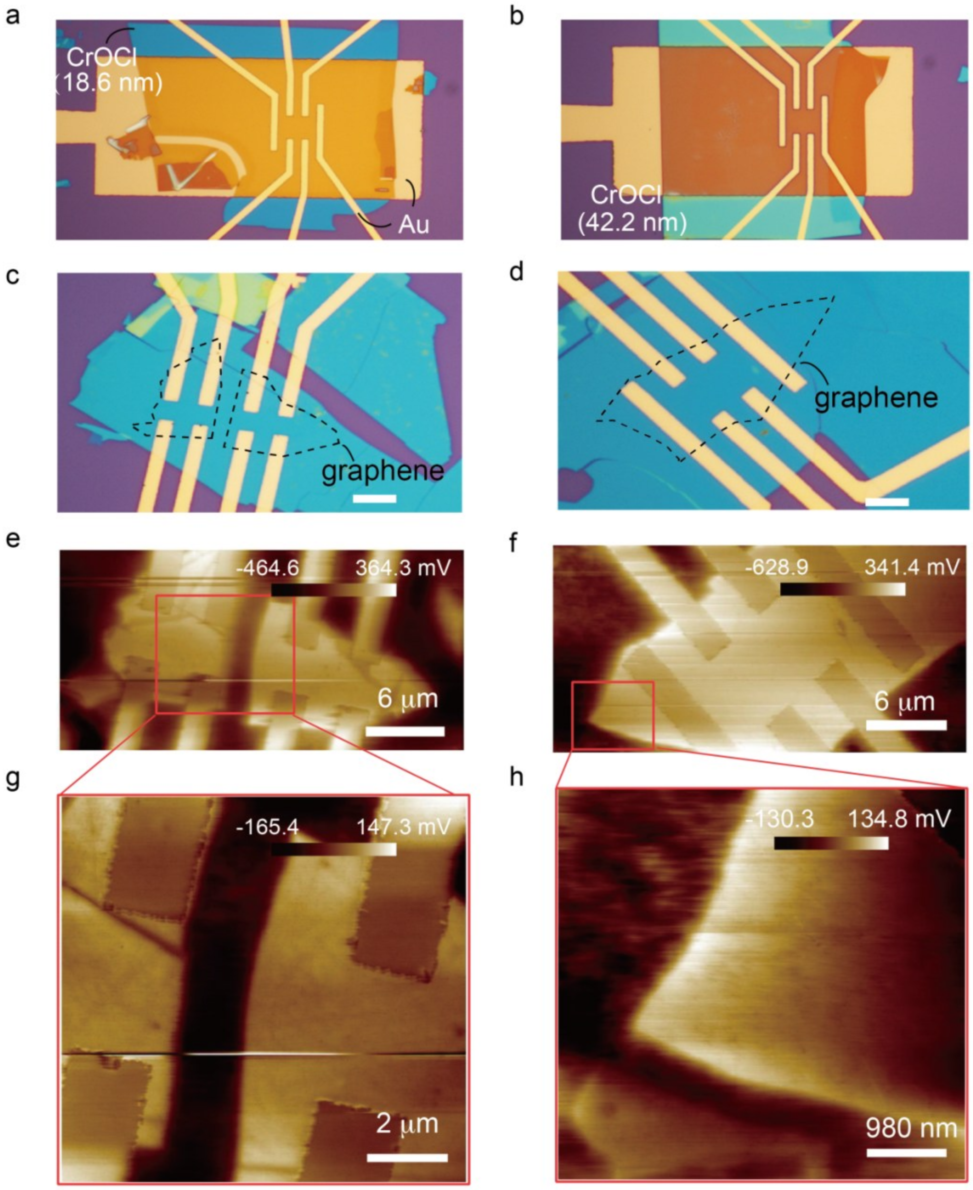

Supplementary Figure 2. **Characterizations of bulk conductance of CrOCl.** (a)-(b) Optical images of few-layered CrOCl samples vertically sandwiched between Au electrodes with different thickness of CrOCl itself. No measurable conductance is seen in any of them, indicating that the occurrence of weak bulk conductivity in thin CrOCl requires a surface contact with graphene/graphite. (c) and (d) Optical images of graphene/CrOCl heterostructures without top BN. Scale bars are 5 μm. (e)-(f) Kelvin Probe Force Microscopy (KPFM) images of (c) and (d), with the zoomed area shown in (g) and (h). It is seen that the electric potential is duplicating the morphology of graphene, indicating no in-plane diffusion of conductance in CrOCl. Further studies are needed to elucidate the mechanism of CrOCl bulk conductivity. In this work, we focus on the physical properties at low temperature, where the thin CrOCl is well insulating.

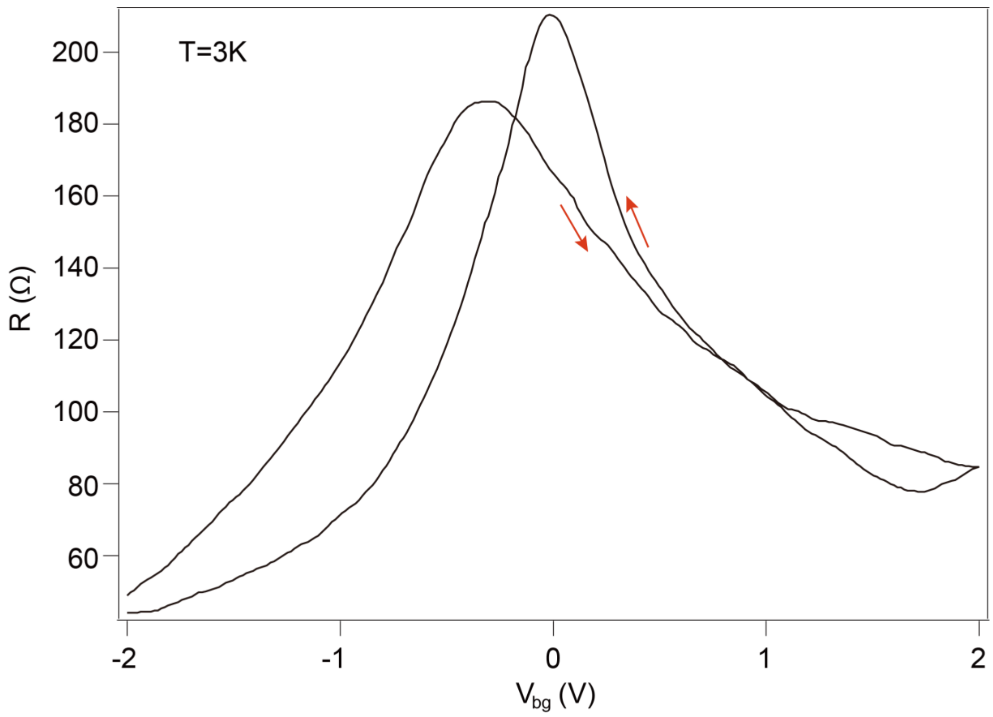

Supplementary Figure 3. **Field effect curves of a typical CrOCl/graphene/h-BN device at *T*= 3 K with a single graphite back gate.** Hysteresis can be seen in the trace and re-trace curves recorded, while there seems to be a Dirac point as in usual graphene since there is a resistance maxima. However, further analysis show that the carriers in this whole gate range are constantly hole-type. Only displacement fields can bring the system into the regime of electron-dominated transport.

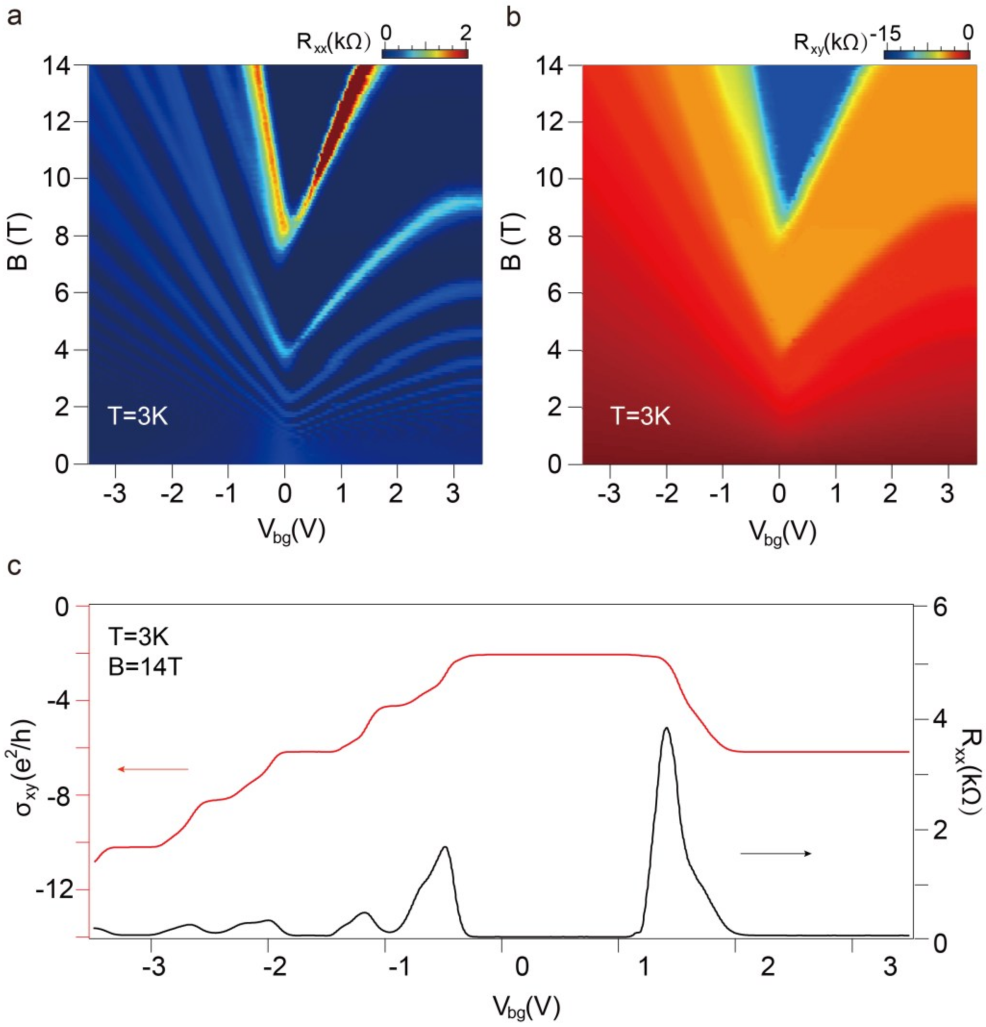

Supplementary Figure 4. **Field effect curve in different magnetic fields in a typical CrOCl/graphene/h-BN device with a single bottom graphite gate beneath the CrOCl.** (a) and (b) are color maps of R_{xx} and R_{xy} in the parameter space of V_{bg} and B, respectively. (c) Line profile of R_{xx} and σ_{xy} in color maps at 14 T and 3 K. It is seen that σ_{xy} is always hole-type, with quantized conductance at negative filling fractions in the whole gate range.

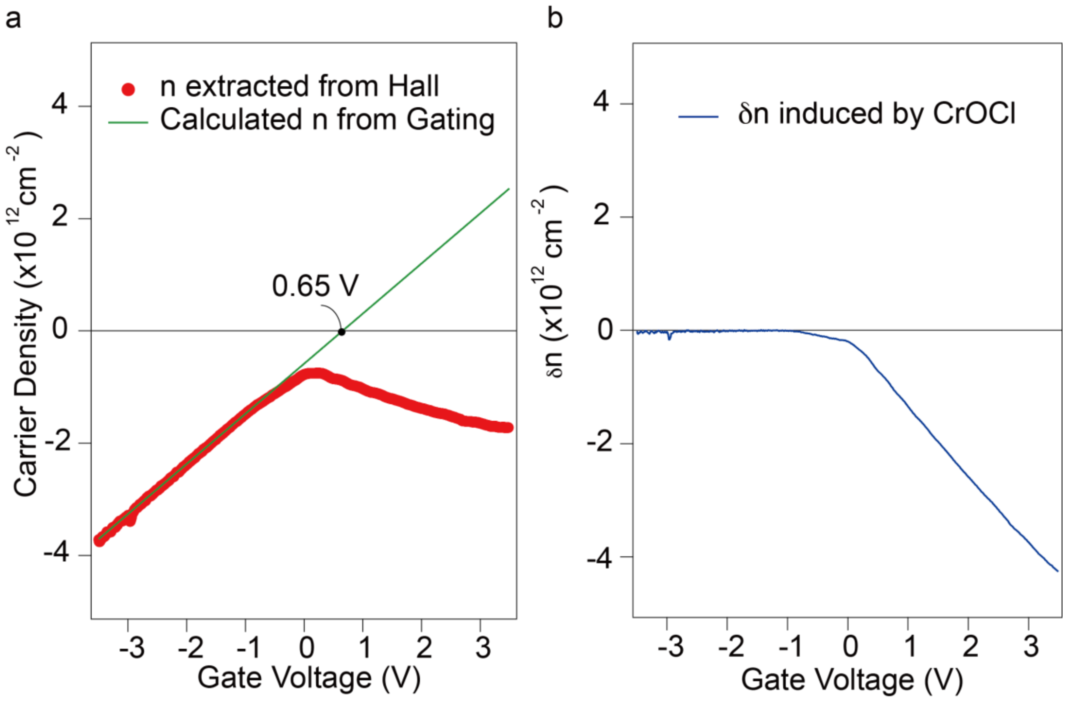

Supplementary Figure 5. **Carrier density in a typical CrOCl/graphene/h-BN device with a single bottom graphite gate beneath the CrOCl.** (a) Charge carrier density as a function of gate voltage. The solid red circles denote *n* measured from linear R_H in low magnetic fields (before the quantum oscillations start, *i.e.*, B < 0.5 T). The green solid line is a linear fit of the left part of the experimental data. (b) Dependence of $\delta n = n_{\text{fitted}} - n_{\text{Exp}}$ and gate voltage. The δn is a measure of the number hole-type carriers induced by charge transfer from CrOCl to graphene, which is actually excessive to the gate induced electrons via capacitive coupling from the gate voltage.

2. Scanning tunneling microscope morphology of monolayered graphene on few-layered CrOCl

As shown in Supplementary Figure 6, scanning tunneling microscopy (STM) characterizations suggest rather clean morphology of graphene lattice. And such single-gated graphene on CrOCl samples show strong hole doping with the Fermi level shifted downwards by about 200 meV.

Scanning tunneling spectra (STS) of the sample are also measured. Shown in Supplementary Figure 6c, the differential conductance as a function of bias voltage is measured at B=10 T and T=4 K. One can see that at a bias lower than 0.2 V (hole doping of ~200 meV in energy), conventional LLs of graphene is developed, with the Nth peak voltage fitted by a linear relation of E~$N^{1/2}$, shown in (d). Electron side LLs are not well developed in the STS, and due to the lack of working gate, we could not have a gate tuning STS in the tested samples. And, since there is no possibility for a top gate, the general features observed in transport in dual gated samples cannot be obtained in STM studies.

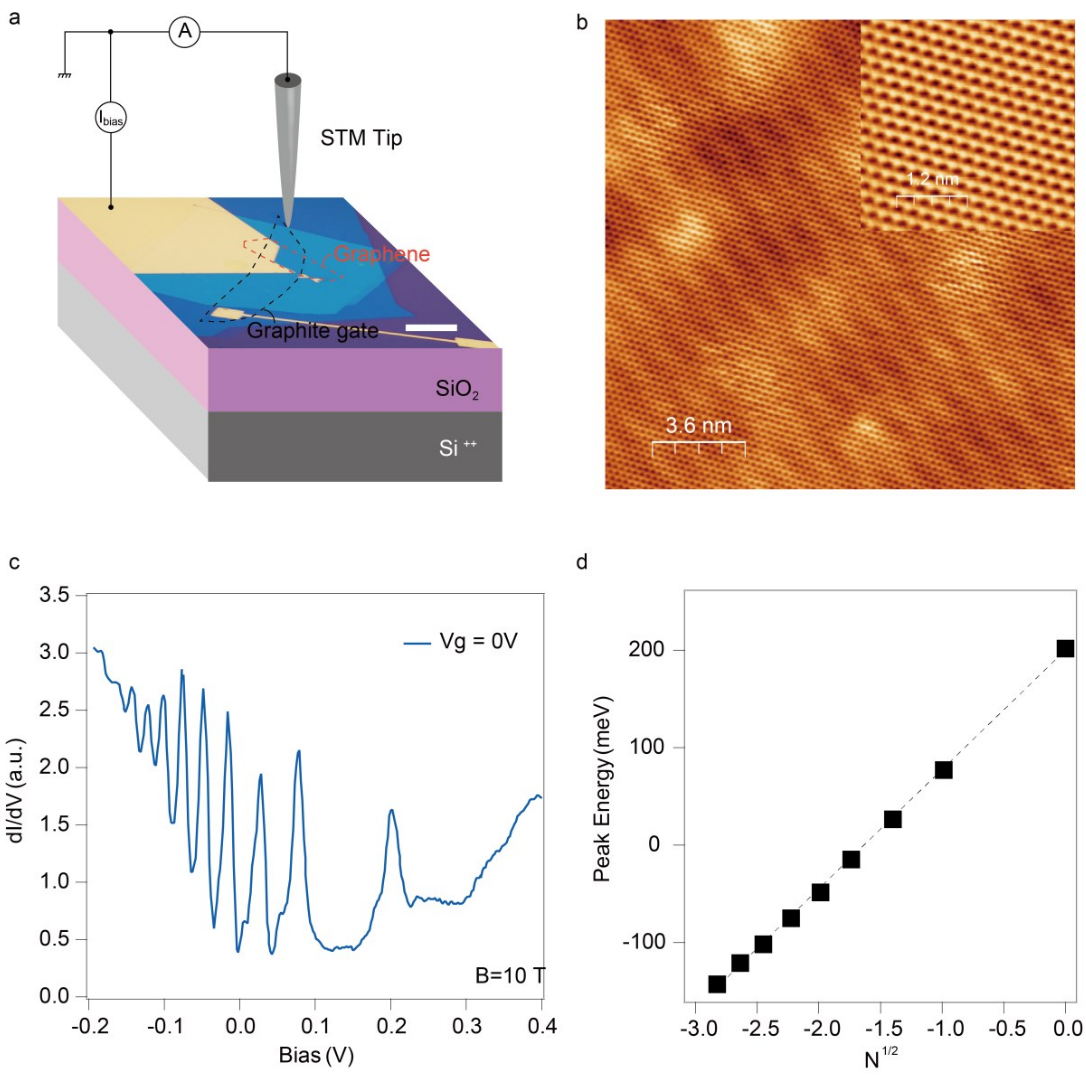

Supplementary Figure 6. **Scanning tunneling microscopy study of Graphene/CrOCl heterostructure**. (a) Schematic picture of the experimental setup. Monolayer graphene was placed on top of few layered CrOCl. (b)Atomic resolution morphology of the graphene/CrOCl heterostructure. Inset shows the honeycomb lattice of graphene, while long range lines of the moiré pattern can be seen. (c)

3. Characteristic of dual gated graphene/CrOCl heterostructures

When a finite perpendicular magnetic field *B* is applied, LLs develop in graphene, giving rise to a stripe-like features along constant filling fractions in the dual gated mapping of the field-effect curves, and usually symmetric with respect to the charge neutrality in conventional graphene samples.[1,2]

However, in the h-BN/graphene/CrOCl heterostructures with dual gates, the LLs become much different when entering certain gate ranges, yielding a new phase exhibiting cascades-like LLs in general, as illustrated in the schematic phase diagram in Supplementary Figure 7. This phenomenon is reproducible in multiple monolayered graphene samples stacked on different thicknesses of CrOCl flakes, and is verified by sweeping along different directions within the reachable gate range (Supplementary Figure 8), which thus rules out significant distortions from possible gate-hysteresis in the system.

---

1 Maher, P. *et al.* Tunable fractional quantum hall phases in bilayer graphene. Science 345, 61–64 (2014).
2 Li, J. *et al.* Even-denominator fractional quantum hall states in bilayer graphene. Science 358, 648–652 (2017).

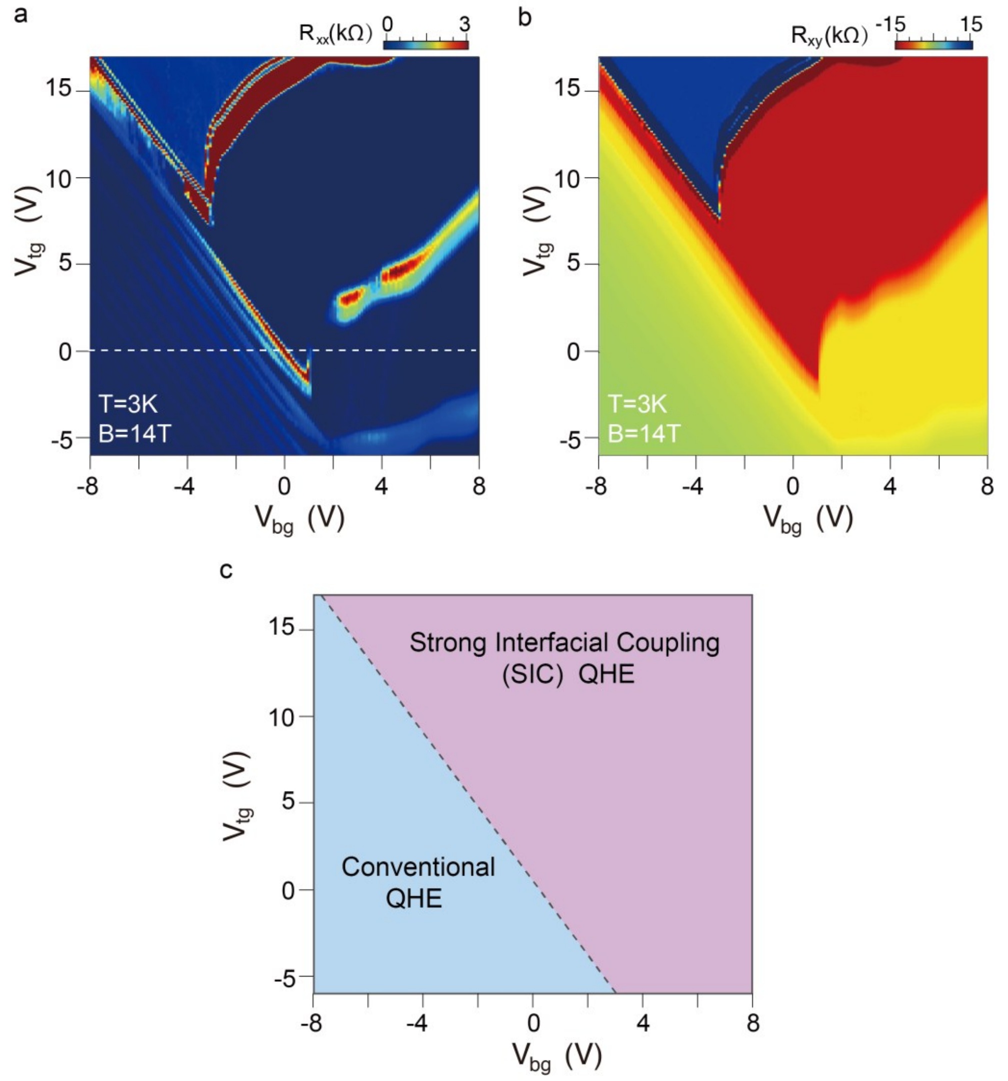

Supplementary Figure 7. **Color map of channel resistance in a typical dual-gated Au/CrOCl/graphene/h-BN/Au sample.** (a) and (b) are color maps of a dual gate scan of field effect measured at T=3 K and B=14 T. (c) A schematic phase diagram showing roughly two distinct areas of conventional QHE and SIC-QHE, whose behaviors of Landau quantization are totally different. A more detailed phase boundary between the two phases is discussed in Fig. 4a-b in the main text, and in the Supplementary Note 1. It can be seen that results in Supplementary Figure 5 can be regarded as a line cut along V_{tg}=0 in the dual gated map, such as that along the dashed white line in Supplementary Figure 7a. Electron carriers dominated transport can be realized at a very large displacement field, at the order of about 0.8 V/nm or higher, indicated by the positive R_{xy} (blue color region) in (b).

4. Reproducibility of the observed exotic phenomenon in multiple samples

Devices	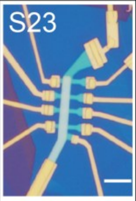 S23	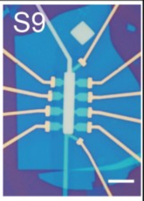 S9	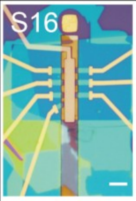 S16	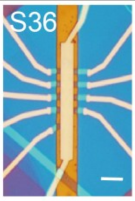 S36	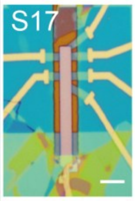 S17	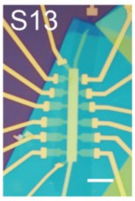 S13	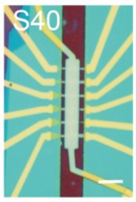 S40
h-BN	32.6 nm	28.2 nm	27.4 nm	25.1 nm	29.8 nm	31.3 nm	44 nm
CrOCl	13.5 nm	19.4 nm	21.3 nm	24.4 nm	26.7 nm	34.3 nm	38 nm

Supplementary Figure 8. **Summarization of samples with various layer thicknesses.** Samples fabricated with different thicknesses of top h-BN and bottom CrOCl layers are listed in the table. It is seen that with thicknesses of CrOCl ranging from 13.5 to 38 nm.

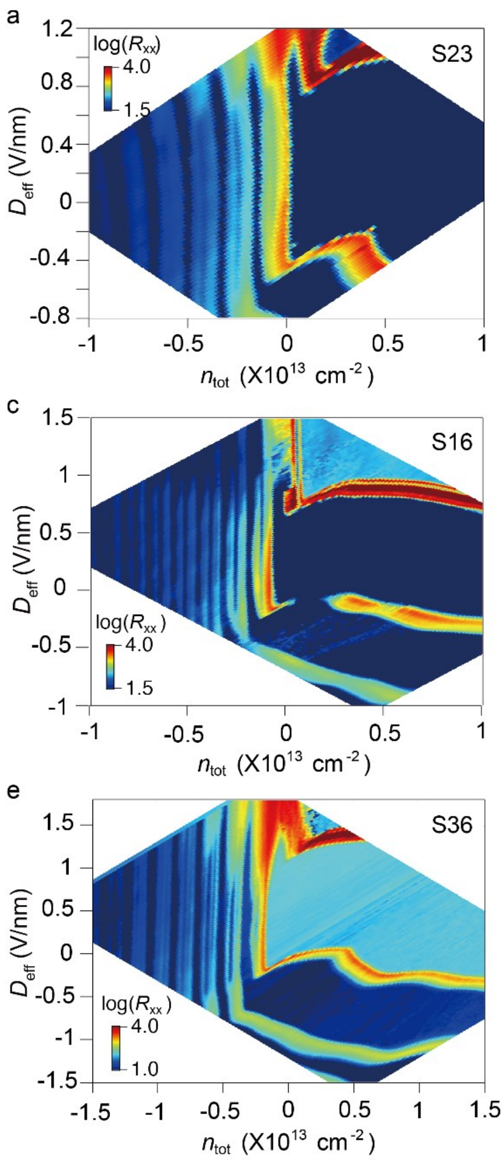

Supplementary Figure 9. **Channel resistance R_{xx} in the D_{eff}-n_{tot} space at 12 T for different samples tested.** (a)-(f) shows the channel resistance (T = 3 K and B = 14 T for Device-S13, S16, S17, and S23; T = 1.5 K and B = 12 T for Device-S36 and S40) in the D_{eff}-n_{tot} space. Thicknesses of h-BN and CrOCl for each sample are indicated in Supplementary Figure 8. It is seen that the general behaviors of those samples are rather consistent.

5. Hysteresis analysis of the dual gate scans by sweeping the gate voltages along different directions in Device-S13

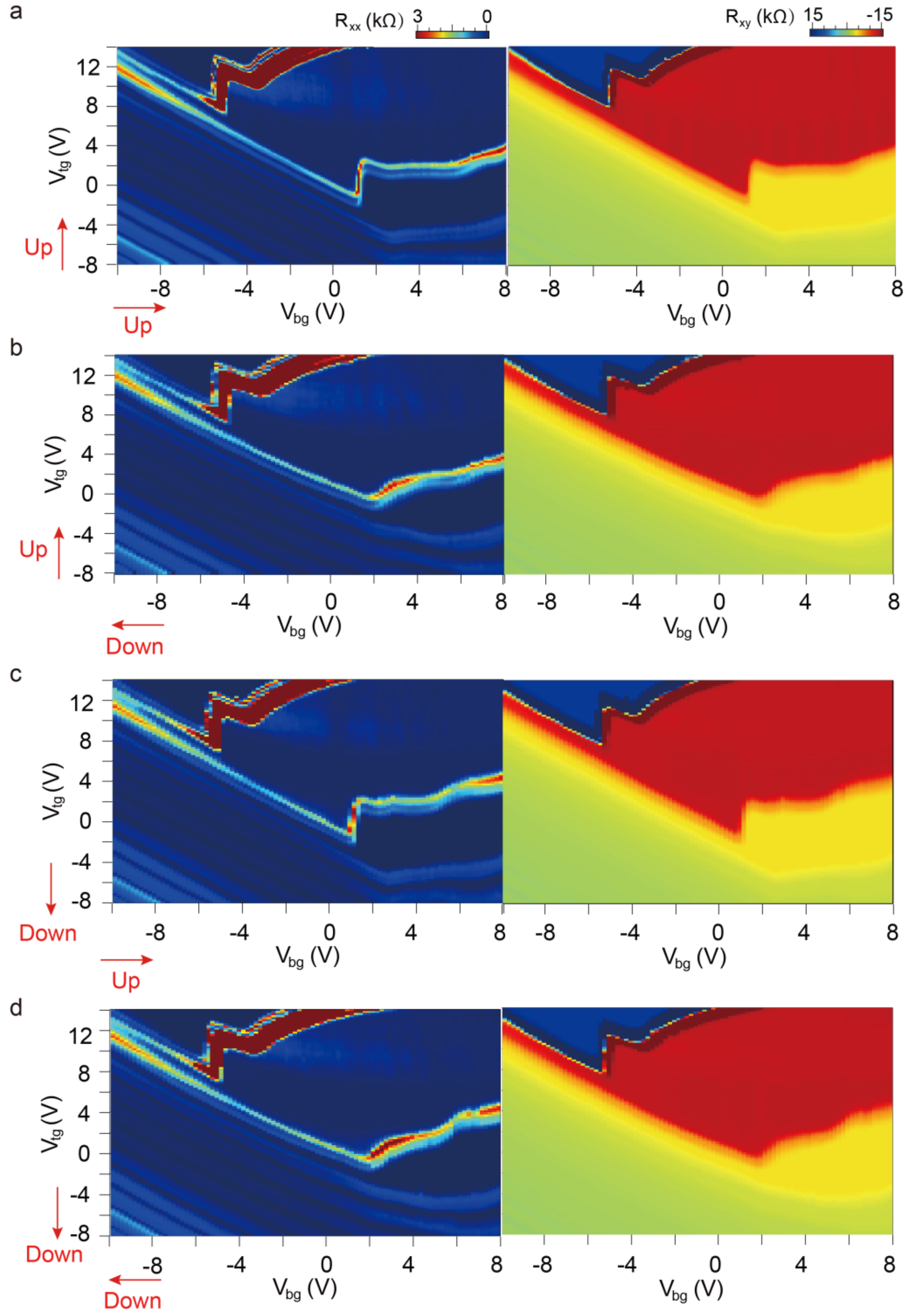

Supplementary Figure 10. **Hysteresis analysis of the dual gate scans.** Data of R_{xx} and R_{xy} measured at T= 3 K and B=14 T for Device-S13 are shown by sweeping along different gate directions as indicated by the red arrows. Slight hysteresis exists depending on gate history but general behaviors of the Landau quantization are the same.

6. Plotting the dual gated map of channel resistance into the space of D_{eff}-n_{tot}

The way we obtained n_0 and D_0 is given in Supplementary Figure 11. With these two values, the sample resistance scanned in parameter space of V_{tg} and V_{bg} can be transformed into n_{tot}-D_{eff} coordinates, with n_{tot}=0 calibrated to ν=0 Landau quantization in the conventional QHE phase, as given in the example in Supplementary Figure 11.

By measuring the Landau plateaus at high field (14 T in this example) along a line cut with fixed D_{eff} (red dashed line in (a), D_{eff}=0.35 V/nm), and to extrapolate the quasi-linear relationship to the ν=0, one gets a V_{tg0} (and hence V_{bg0}, as they are in the line of fixed D_{eff}) indicated by the black arrow in Supplementary Figure 11b. Because all extrapolations of the above operation at any D yield the same n_0 due to the conventional nature of LLs, and thus the residual doping n_0 is defined as the white dashed line in Supplementary Figure 11a. One can then re-plot the Supplementary Figure 11a into a parameter space of n_{tot} and D, using $n_{tot} = (C_{tg}V_{tg} + C_{bg}V_{bg})/e - n_0$ and $D = (C_{tg}V_{tg} - C_{bg}V_{bg})/2\varepsilon_0 - D_0$.

Notice that D_0 (residual displacement field) in principle should be in the charge neutrality as well. However, since D_{eff} is a relative value, we chose the D_0 in our study by using the point where V_{tg}=0 V in the line of n_0 (i.e., in the white dashed line) for simplicity, as shown in Supplementary Figure 11a. Thus, the dual-gated mapping is transformed into n-D mapping, as shown in Supplementary Figure 11, with the obtained white dashed line to be at the 'zero' in the axis of n_{tot}.

For determining the capacitances C_{tg} and C_{bg} of top and bottom gate dielectric, we first, the slope k of LLs in the color map at high fields (the magnetic fields under which there is observable Hall quantization in R_{xy}) is one relation we can take advantage. Along the LL, the filling fraction (i.e., the carrier concentration) is fixed by definition. Therefore, one can measure the slope k of an arbitrary LL in the dual gated map (the lower-left part of (a), parallel to the white dashed ν=0 line). One obtains $k=dV_{\mathrm{tg}}/dV_{\mathrm{bg}}=C_{\mathrm{tg}}/C_{\mathrm{bg}}$, as given by $n_{\mathrm{tot}} = (C_{tg}V_{tg} + C_{bg}V_{bg})/e - n_0$. Then, we obtain the n_{tot}, which is by measuring the Landau index $\nu = -\frac{nh}{eB}$. Therefore, one can obtain the exact filling fraction by picking each quantized plateau, which follows a quasi-linear relation versus one of the two gate voltages. The two capacitance C_{tg} and C_{bg} of each gate dielectric can then be solved using the above two sets of experimental values (slope k and filling fraction ν), using the equation of $n = (C_{tg}V_{tg} + C_{bg}V_{bg})/e - n_0$. In our measured samples, we noticed that, by measuring the slope of $V_{\mathrm{TG}}/V_{\mathrm{BG}}$ at fixed n, dielectric constant of CrOCl at low temperature is estimated to be ~1.4 times higher

than that of h-BN.

Using the same method, one can also plot such color-map at B=0, as shown in Supplementary Figure 13. It is clear that Dirac-like peak still exist but in an unusual manner, with the charge neutrality indicated by the white dashed line in Supplementary Figure 13.

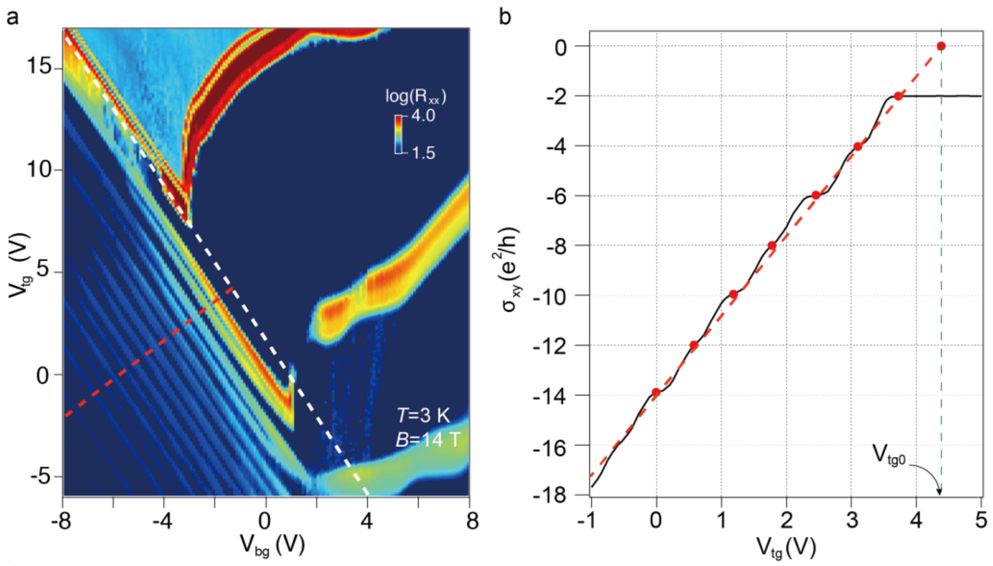

c

$\upsilon(e^2/h)$	0	-2	-4	-6	-8	-10	-12
V_{tg}(V)	4.301	3.726	3.088	2.424	1.766	1.161	0.589
V_{bg}(V)	-1.364	-1.672	-2.014	-2.369	-2.721	-3.044	-3.351
n(cm^{-2})	9.95×10^{11}	-6.77×10^{11}	-1.35×10^{12}	-2.03×10^{12}	-2.71×10^{12}	-3.38×10^{12}	-4.05×10^{12}

Supplementary Figure 11. **Extraction of n_0 from experimental data at high fields (14 T).** (a) Dual gate color map of sample resistance R_{xx} measured at 14 T and 3 K. (b) Filling fraction of Landau quantization obtained in the corresponding R_{xy} curve, with the gate values and filling fractions calculated using ν=nh/(Be), listed in the table in (c). Red value is the n_0 extrapolated from (b).

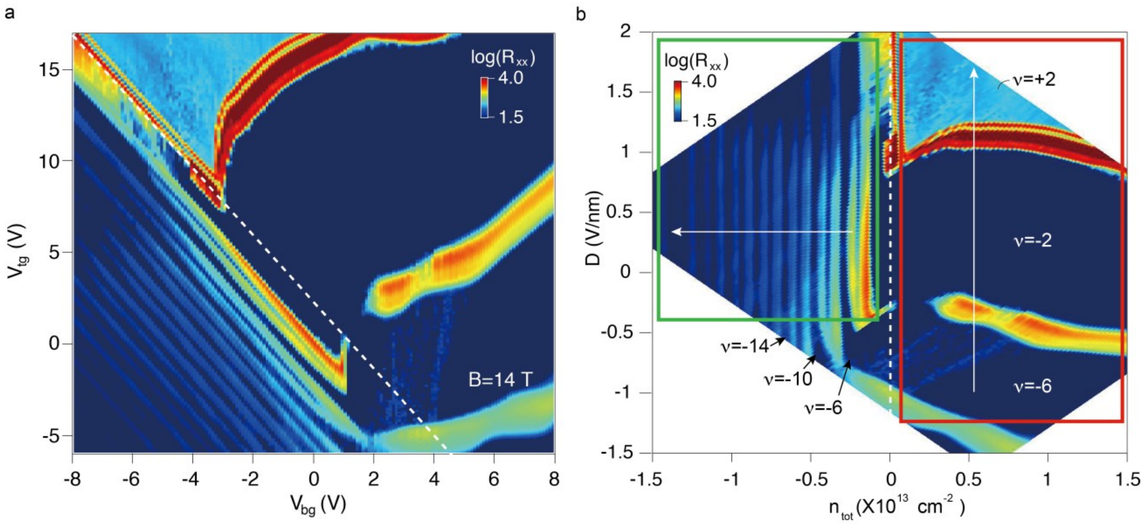

Supplementary Figure 12. **Color maps of sample resistance scanned in dual gates.** (a) The color map of sample resistance scanned in the parameter space of V_{tg} and V_{bg}, at B=14 T and T=3 K. (b) data in (a) plotted in the parameter space of n_{tot} and D. White dashed lines denote n_{tot}= 0. Two regions can be clearly distinguished: the green and red boxed areas. It is obvious that in the green boxed area, a 'conventional' graphene behavior is observed, with the Landau level (LL) quantizations tuned along the axis of n_{tot}, as indicated by the horizontal white arrow, while D has little effect on the LLs. Surprisingly, in the red boxed area, total doping defined by the two gates cannot tune any longer the LLs, but D can now shuffle the Landau quantization, as indicated by the vertical white arrow. This behavior of gate-tunable transition between 'conventional' and 'exotic' QHE is totally new, and has no analogue in the conventional studies of graphene with Dirac peak shifted by charge doping. Detailed discussions can be found in the main text.

7. Carrier doping in dual gated h-BN/graphene/CrOCl device in the space of D_{eff}-n_{tot}

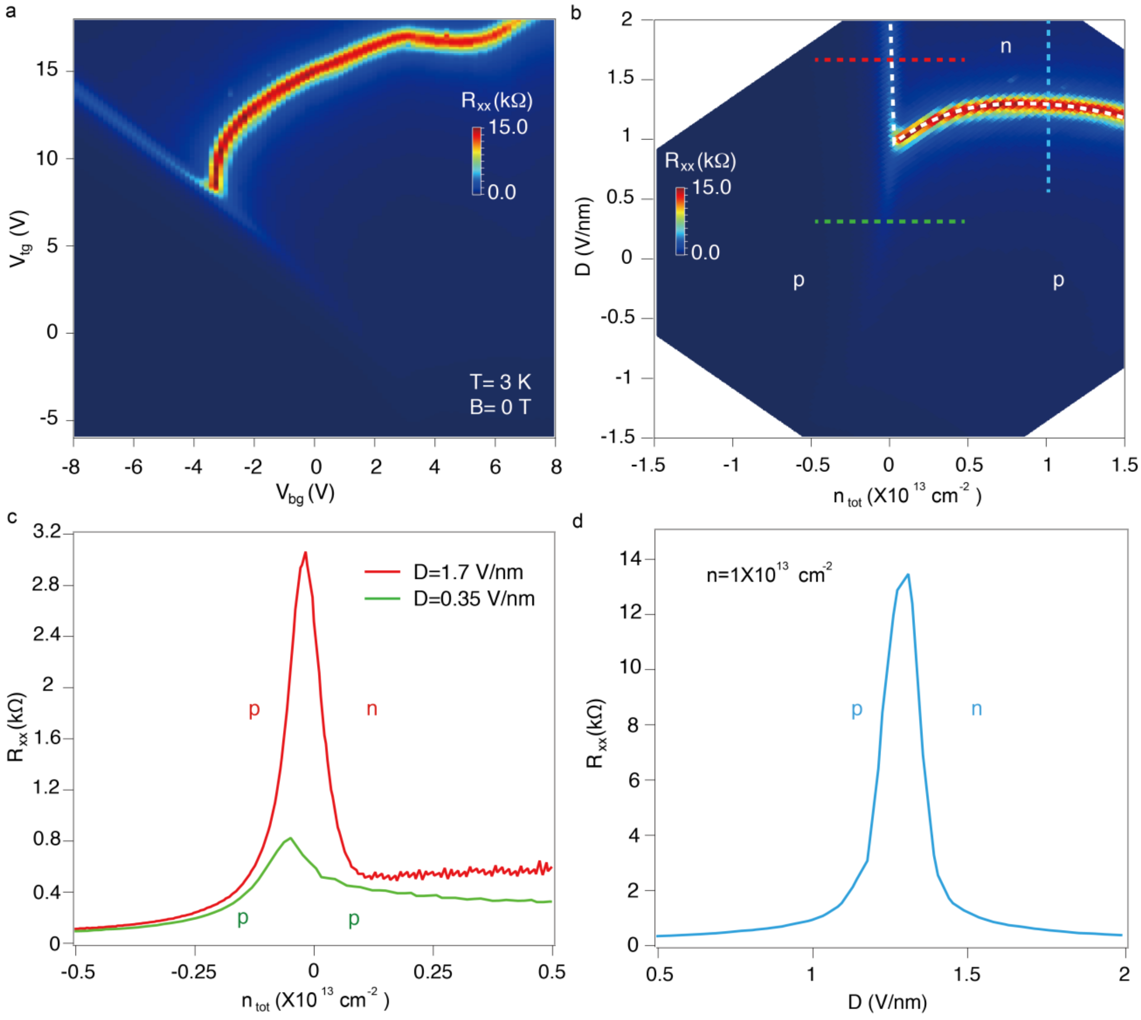

Supplementary Figure 13. **Color maps of typical sample resistance at zero field at 3 K.** (a) The color map of sample resistance scanned in the parameter space of V_{tg} and V_{bg}, at B=0 T and T=3 K. (b) data in (a) plotted in the parameter space of n_{tot} and D. White dashed lines denote the charge neutrality of graphene, where electrons and holes switch signs. (c) Line profile of R_{xx} at fixed D of 0.35 V/nm and 1.7 V/nm, for green and red curve, respectively. Curves are cut along the green and red dashed lines in (a). (d) Line profile of R_{xx} at fixed $n_{tot} = 1 \times 10^{13}$ cm^{-2}, along the blue dashed line in (b). Line profiles of R_{xx} at fixed D of 0.35 V/nm (green) and 1.7 V/nm (red), show some Dirac-peak like behavior, but only the red curve (D=1.7 V/nm) has a sign change from p- to n-type of its carriers, while the green one does not change sign and remained hole-doped throughout its n_{tot} range. The curve in (d) also has a sign change since it crosses the charge neutrality.

8. Hole mobility in a typical dual-gated Au/CrOCl/graphene/h-BN/Au device

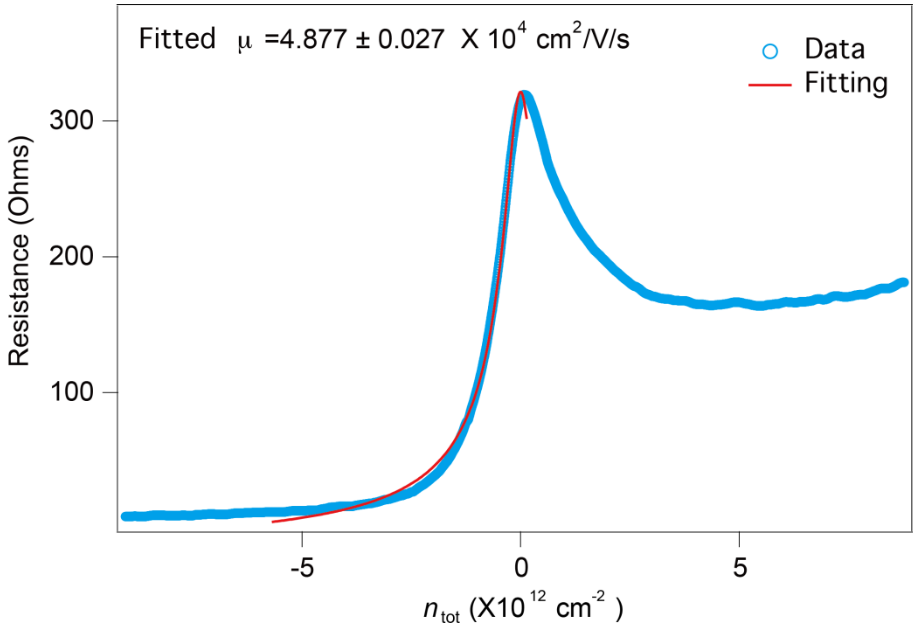

Supplementary Figure 14. Hole mobility in a typical dual-gated Au/CrOCl/graphene/h-BN/Au device. The field effect curve is fitted using the phenomenal formula $R=1/[e\mu*(n_0^2+n^2)^{1/2}]+R_c$, where e is the electron charge, μ is the mobility, n_0 is the residual electron-hold puddle doping, and the fitting parameter R_c denotes the contact resistance. Here, we used only the hole part to fit the curve, since electron side are actually disturbed by charge transfer. Mobility at 3 K in a typical device is estimated to be around 40,000 ~ 50,000 cm^2/V/s, comparable to those reported high quality graphene, yet relatively lower than best mobility reported in h-BN sandwiched graphene devices.

9. Line profile of $n_{graphene}$ obtained from Hall measurement as a function of n_{tot}, showing the difference between $n_{graphene}$ and n_{tot}

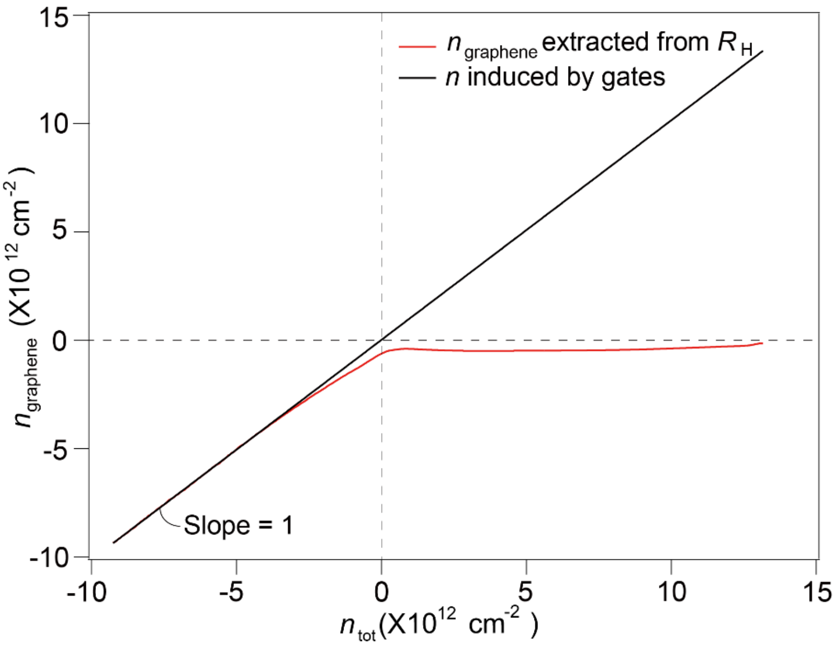

Supplementary Figure 15. Extracting $n_{graphene}$ from Hall resistance at low fields (B<0.5 T). Carrier density n_{eff} in a typical sample at D=0.35 V/nm are extracted from low field Hall effect (linear region of Hall resistance R_H at B<0.5 T), with the n_{eff}= $1/(e^*R_H)$. It is seen that in the conventional phase, gate induced density follows well the n_{eff} extracted from Hall resistance with a slope of ~1, but departures at positive n_{tot}, in agreement with high field measurements at 14 T.

10. A presentation of the "data cube" in the pace of *B*, n_{tot}, and D_{eff} of the currently studied system

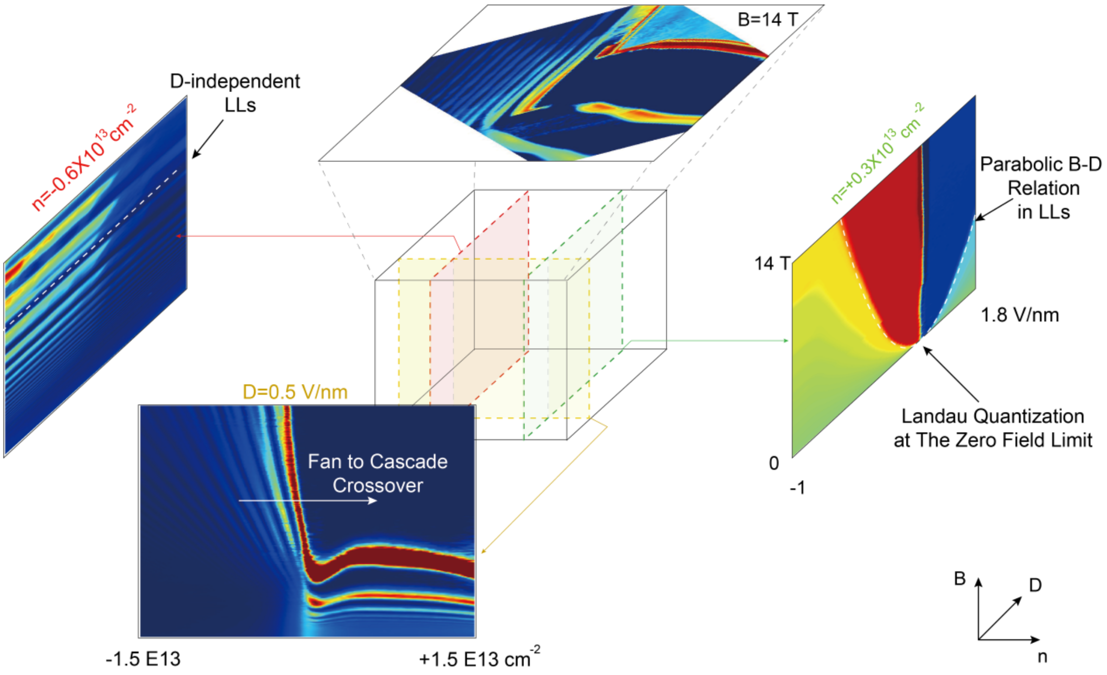

Supplementary Figure 16. **The "data cube" of the currently studied system.** Sliced color maps from Fig.2 in the main text are shown in different planes in the *B-D-n* coordinates. For simplicity, the exact numbers in all axes are omitted, with only ranges of them given in the graph. In such a 'data-cube' presentation, all surprising experimental findings are rather straightforward to see, including: 1). the crossover from Fan- to Cascade-like Landau quantization; 2). the parabolic *B-D* relation in LLs; and 3). the very surprising zero field limit of the quantized plateau.

11. Control samples showing similar data to the one in Fig. 2 in the main text

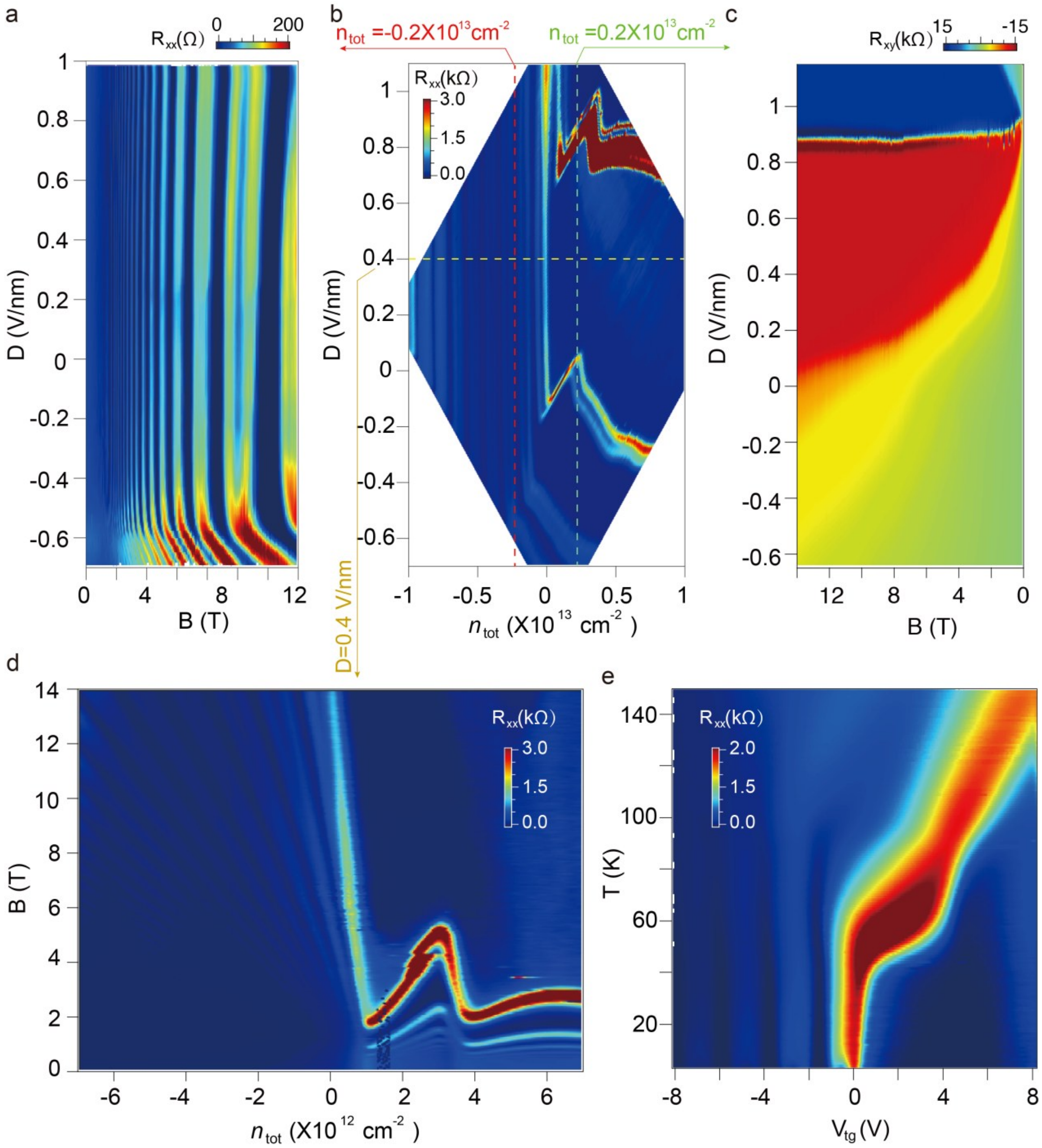

Supplementary Figure 17. **Reproducibility of gate tunable charge transfer in the quantum Hall regime in Device-S13.** (a) and (c) are color maps of R_{xx} and R_{xy} as a function of magnetic field B. Date recorded along dashed lines in (b). (b) R_{xx} in the parameter space of D and n, plotted from Supplementary Figure 9b. (d) Gate tunable cross over from fan-like to cascades-like Landau quantization. (e) R_{xx} in the parameter space of temperature and top gate at 14 T. QHE is seen to be prevailing up to 100 K.

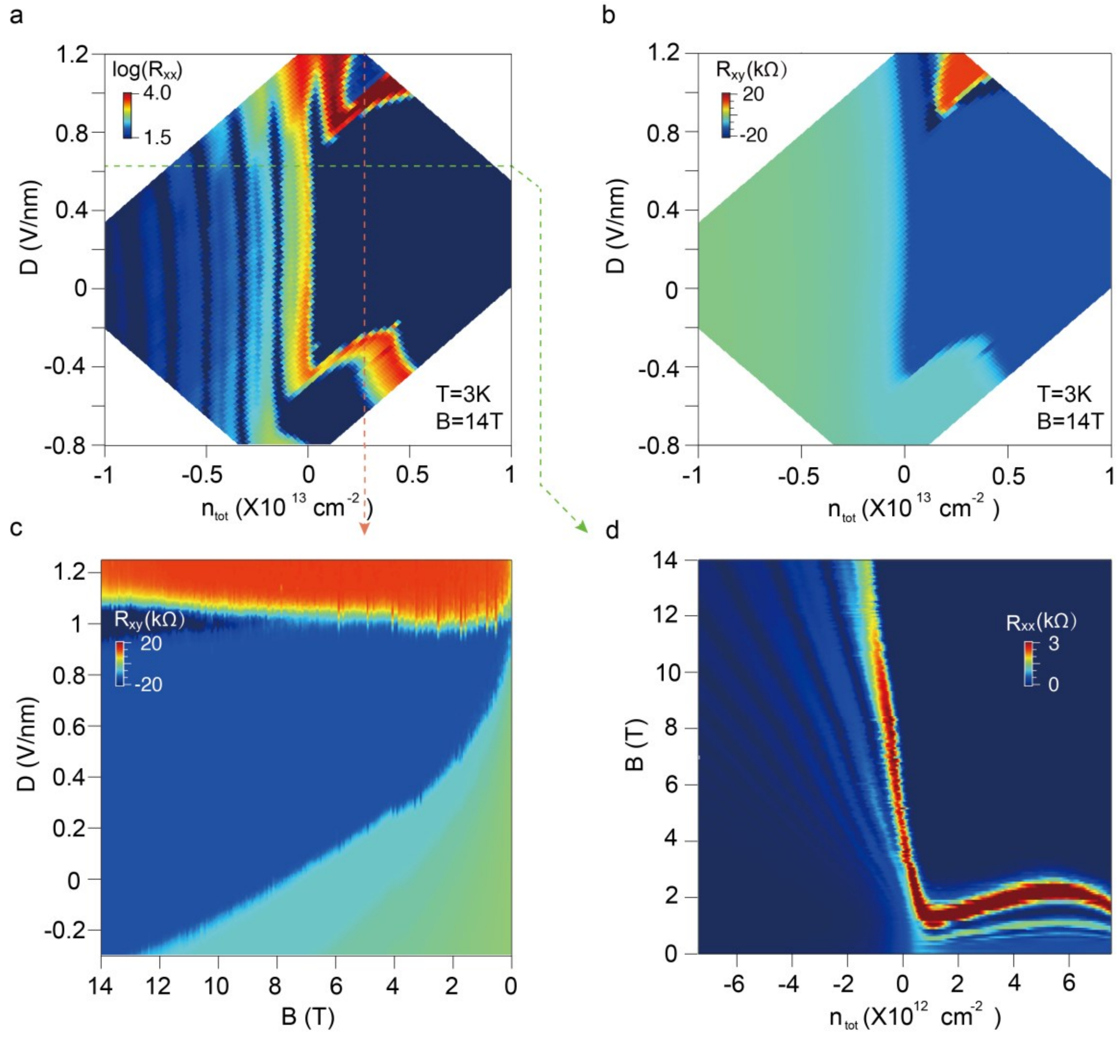

Supplementary Figure 18. **Reproducibility of gate tunable charge transfer in the quantum Hall regime in Device-S23.** (a)-(b) R_{xx} and R_{xy} in the parameter space of D and n, measured at 14 T and 3 K. (c) Color map of R_{xy} at fixed D as a function of magnetic field B. Data recorded along the red dashed lines in (b). (d) Gate tunable cross over from fan-like to cascades-like Landau quantization at D=0.62 V/nm, obtained by scanning B along green dashed line in (a). General behavior is similar as compared to the sample reported in the main text.

12. Data obtained in typical sample fabricated in a glove box

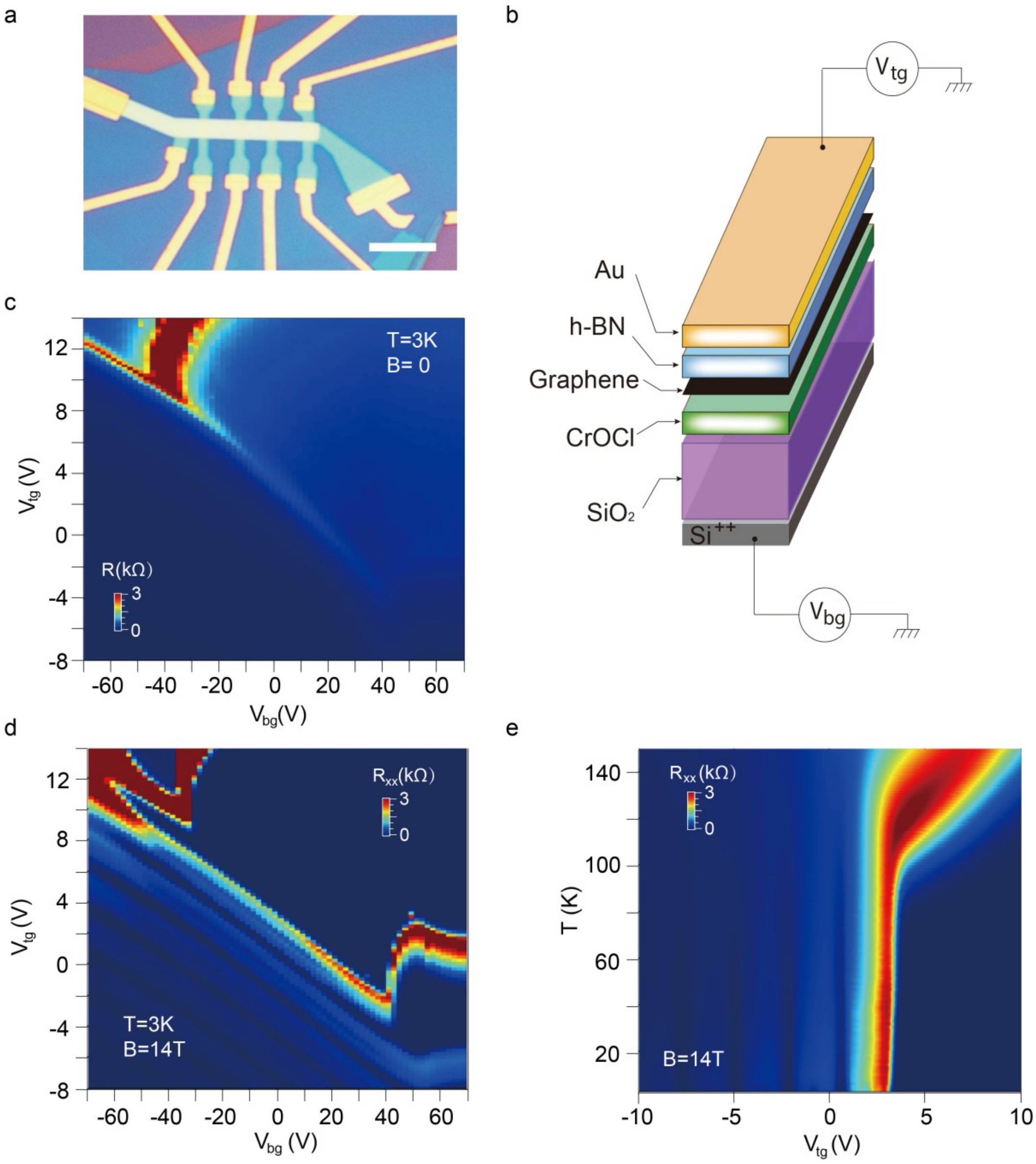

Supplementary Figure 19. **Reproducibility of gate tunable charge transfer in the quantum Hall regime in a control sample fabricated in glove box.** (a) Optical image and (b) art view of a typical h-BN/graphene/CrOCl sample (Device-S23) fabricated in a glove box, without local bottom gate. Top Au gate and remote Si gate are used. (c)-(d) Color maps of a dual gate scan of field effect measured at B=0 T, and B=14T, respectively. Data measured at T= 3 K. (e) R_{xx} in the parameter space of temperature and top gate at 14 T. QHE is seen to be prevailing up to 120 K. Similar behaviors are found as compared to the samples fabricated in air, ruling out possible strong moisture or air adsorption in the interface of graphene/CrOCl heterostructures.

13. Zoomed-in map of channel resistance in the δ*D*-*B* space

In addition to the map in Fig. 3a-b in the main text, we also performed much slower scan with higher resolution of the δ*D*-*B* map in the magnetic field range of ±1 T. Sample Device-40 was examined.

It is seen that, even in the zoomed-in scans in Supplementary Figure 20, there is no coercivity field seen in the quantization field, which is different from the quantum anomalous Hall effect (QAHE) scenario.

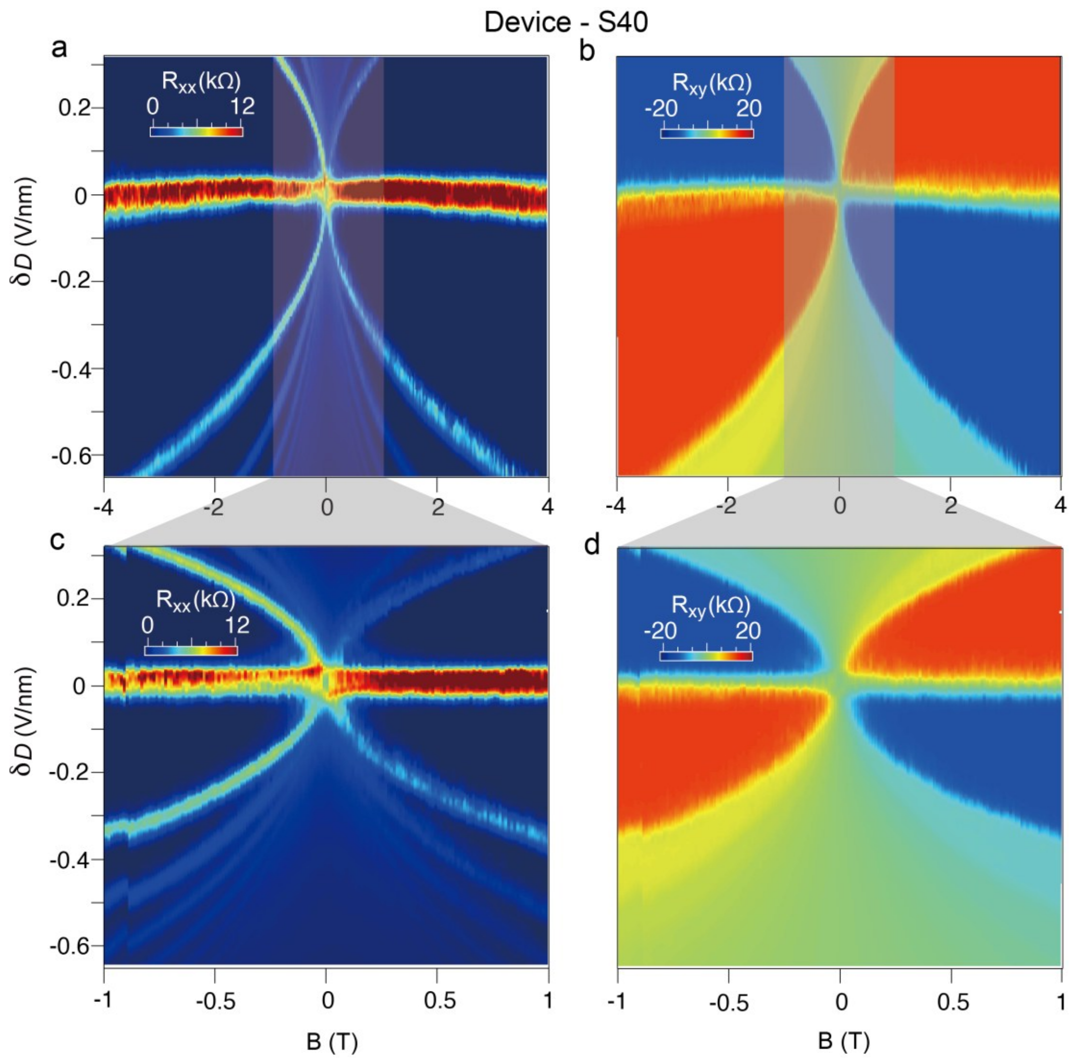

Supplementary Figure 20. Zoomed-in scan of δ*D*-*B* mapping for Device-S40. Data obtained at $T = 1.5$ K and $n_{tot} = 2.5\times10^{12}$ cm^{-2} in Device-S40.

14. Ruling out trivial effect of gate leakage and testing multiple samples in the space of δ*D*-*B* to a maximum temperature before gate leakage takes place

We performed the gate leakage measurements for multiple samples at 1.5 K, which all show negligible leak current at the order of less than hundreds of pA throughout the tested gate range, as shown in Supplementary Figure 21. Few layered CrOCl is proven to be a good dielectric material at low temperatures.

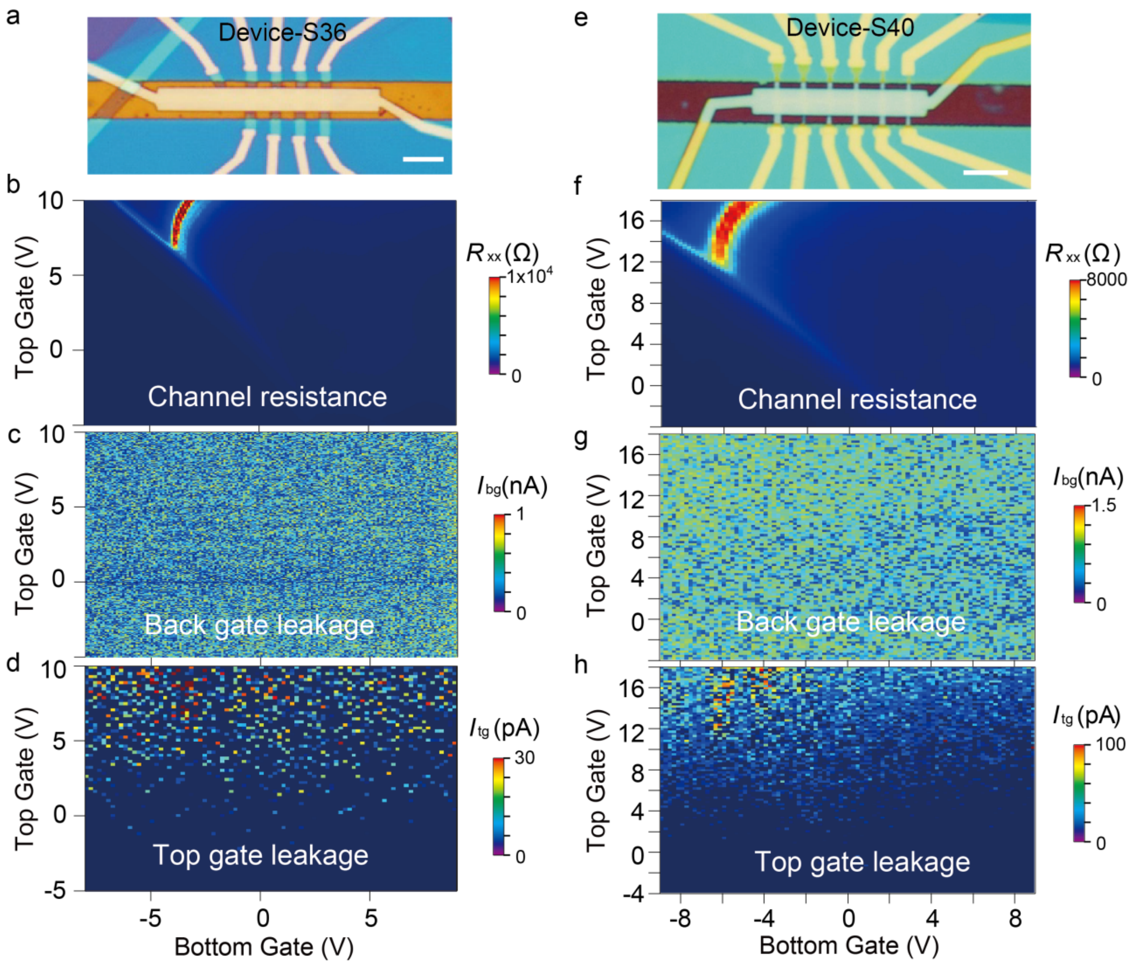

Supplementary Figure 21. Negligible gate leakage currents in two typical devices. Optical image of the sample and the Channel resistance R_{xx} in the space of V_{tg} and V_{bg} are shown in (a)-(b) and (e)-(f), for Device-S36 and Device-S40, respectively. Leakage currents in back and top gates are shown in (c)-(d) and (g)-(h), for Device-S36 and Device-S40, respectively. These data were measured at T = 1.5 K. Scale bars are 5 μm.

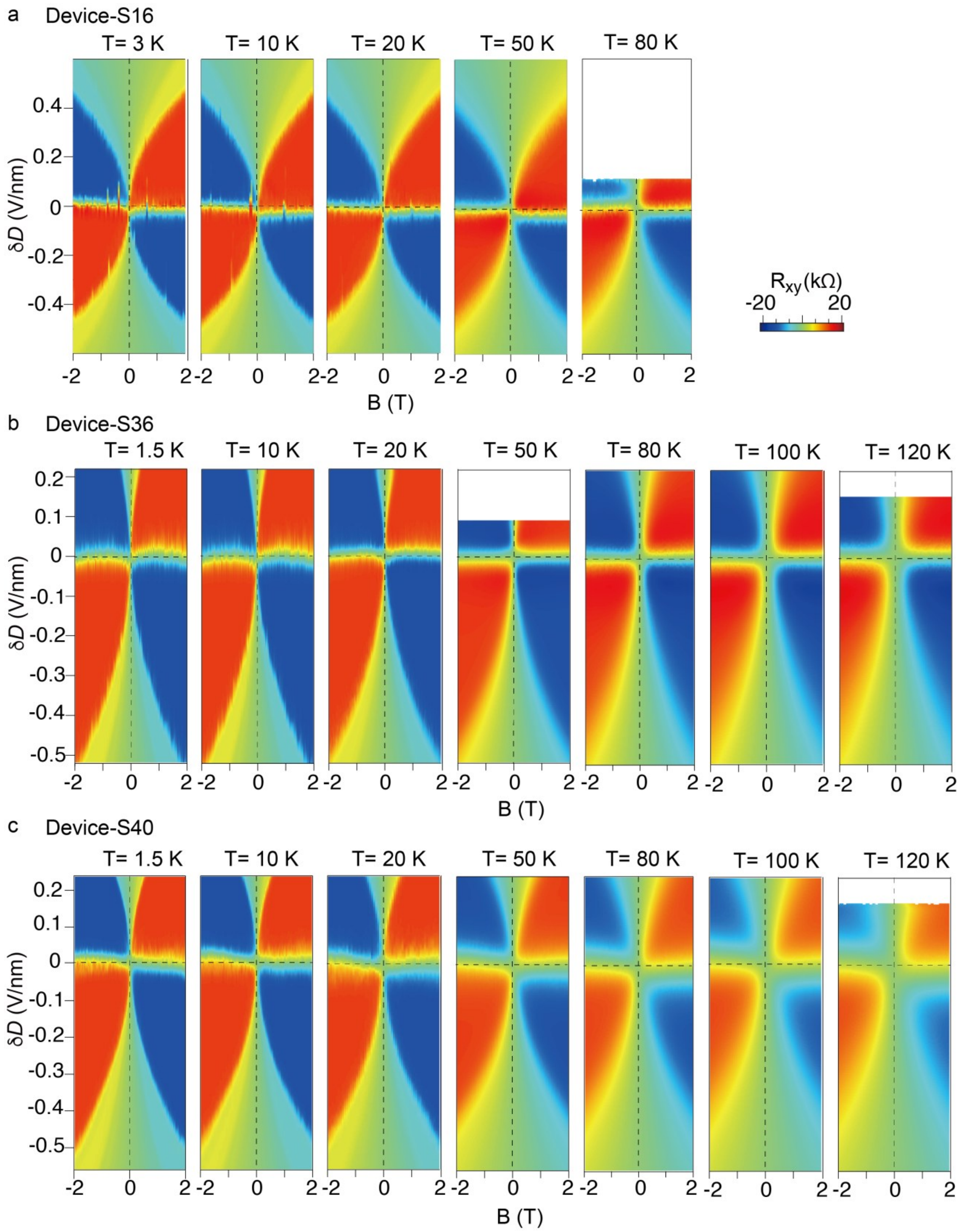

Supplementary Figure 22. Robust Hall quantization in the SIC-Phase. R_{xy} in three typical samples (Devices-S16, S36, and S40) in the space of δD-B, measured at different temperatures, respectively. All images share the same color scale in (a).

We have, while keeping the gate leakage negligible, managed to measure samples (Devices-S16, S36, and S40) at temperatures up to 120 K (higher temperature will lead to gate leakages) the δD-B mapping at fixed n_{tot} (n_{tot} was set to be 2.0×10^{12} cm^{-2}, 2.0×10^{12} cm^{-2}, and 2.5×10^{12} cm^{-2} for Device-S16, Device-S36 and Device-S40, respectively), as shown in Supplementary Figure 22.

Line profiles of R_{xy} in Supplementary Figure 22 are given in Supplementary Figure 23. It is seen that, at finite values of δD (indicated in each panel in Supplementary Figure 23), the quantization may slightly vary from sample to sample, but they all exhibit well quantized ν=±2 plateau below 80 K. Especially, in Device-40, the plateau is still well quantized at B= - 2.5 T and T = 120 K, as can be seen in Supplementary Figure 23c.

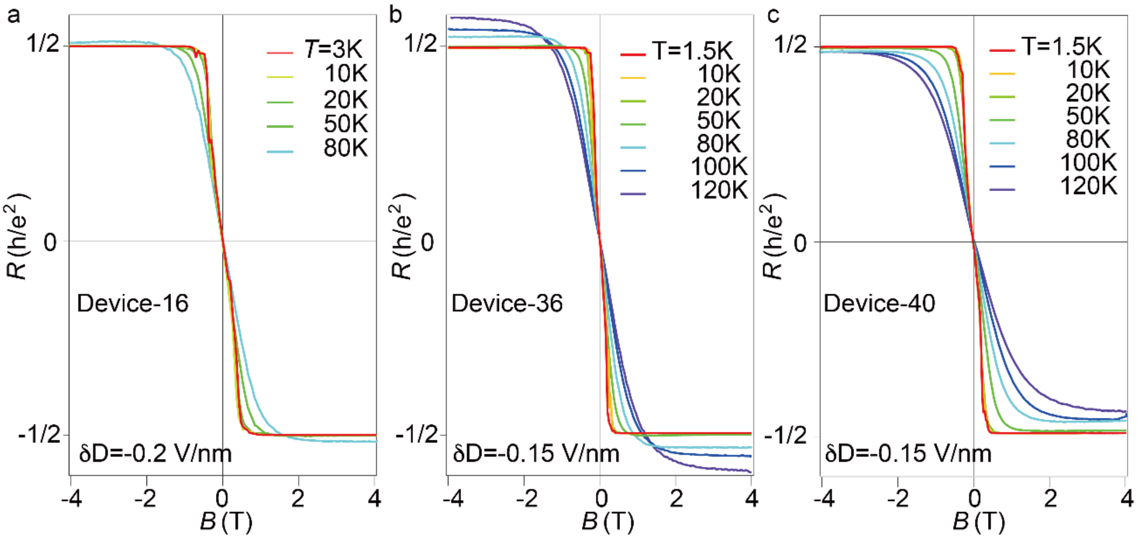

Supplementary Figure 23. Line profiles of R_{xy} at finite δD. (a)-(c) are line cuts from the maps shown in Supplementary Figure 22. Among those tested samples, Device-40 still shows 95 % quantization of $2e^2/h$ (e is elementary charge and h is the Planck constant) at B= - 2.5 T and T = 120 K.

15. Air instability of FeOCl, a sister compound of CrOCl

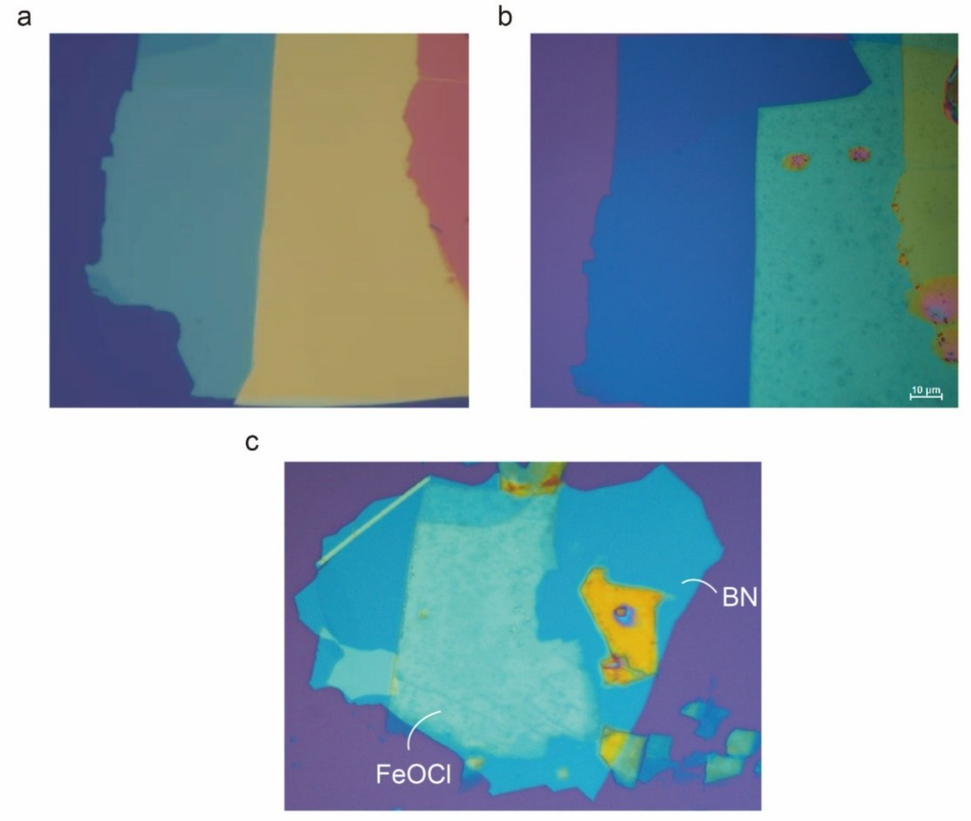

Supplementary Figure 24. **Air instability of FeOCl, a sister compound of CrOCl**. Optical micrograph images of FeOCl before (a) and after (b) annealing in a glove box. (c) Optical micrograph image of FeOCl flake encapsulated by h-BN after annealing in a glove box. Degradation can be clearly seen even protection from air has been avoided as much as possible.

Supplementary Note 1. The electrostatic model of the h-BN/Graphene/CrOCl

To understand the experimentally observed exotic features, we have in the following developed an electrostatic model in the studied system. We will first list the two major unusual experimental observations, as follows:

1. The 'CNP bending' effect in the D_{eff}-n_{tot} mapping, as indicated by the white dashed line in Supplementary Figure 13b.
2. The increasing distance between each iso-doping lines when the system enters deeper into the SIC-phase, as shown in Figure 2b in the main text.

In the following, we will explain the above two major experimental observations using an electrostatic model. In short, the above observed 'CNP-bending effect' and the 'increasing spacing between iso-lines upon entering further in the SIC-phase' are originated from the interfacial coupling between graphene and CrOCl surfaces. Such interfacial coupling invokes two mechanisms:
1) There exists interfacial energy electron states, which can serve as a reservoir of electrons with very large density of states (DOS), but has no free charges hence does not contribute to the transport directly.
2) A band structure reconstruction takes place once the Fermi level of graphene matches the lowest energy of the DOS of the interfacial states, leading to a band structure reconstruction of graphene, with a significant decrease of electron cyclotron mass (*i.e.*, a sharpened slope of the Dirac dispersion).

We assume that an interfacial charge layer exists in the h-BN/graphene/CrOCl heterostructure, which is located in bulk CrOCl below graphene with a distance of d_2, as shown in Supplementary Figure 25a. The whole system can then be simplified into a capacitance model shown in Supplementary Figure 25b, with charge density defined as $\mathrm{n_t, n_1, n_2, n_b}$, and the chemical potential defined as $\mathrm{V_t, 0, V_2, V_b}$. Here, t denotes top, b denotes bottom. Distance of each dielectric layer are then written as $\mathrm{d_1}, \epsilon_1, \mathrm{d_2}, \epsilon_2, \mathrm{d_3}, \epsilon_3$, with the inter-layer electric field written as $\mathrm{E_1, E_2, E_3}$. Moreover, the electron density in graphene and CrOCl layers are defined as σ_1, σ_2, respectively.

Assuming that the interfacial states of graphene/CrOCl is located in the band gap of CrOCl, and is with high DOS and width, but do not contribute to transport. When the graphene is tuned from the hole-side toward the CNP, i.e., Fermi level (E_F) of graphene

is lower than the energy of the lowest value of the interfacial states (Supplementary Figure 25c), only charges at the Fermi surface of graphene are at play in the transport. Hence everything is rather conventional, and the system behaves as 'pristine' graphene. The energy difference between CNP of graphene and the lowest energy of DOS of the interfacial states is defined as Δ_E.

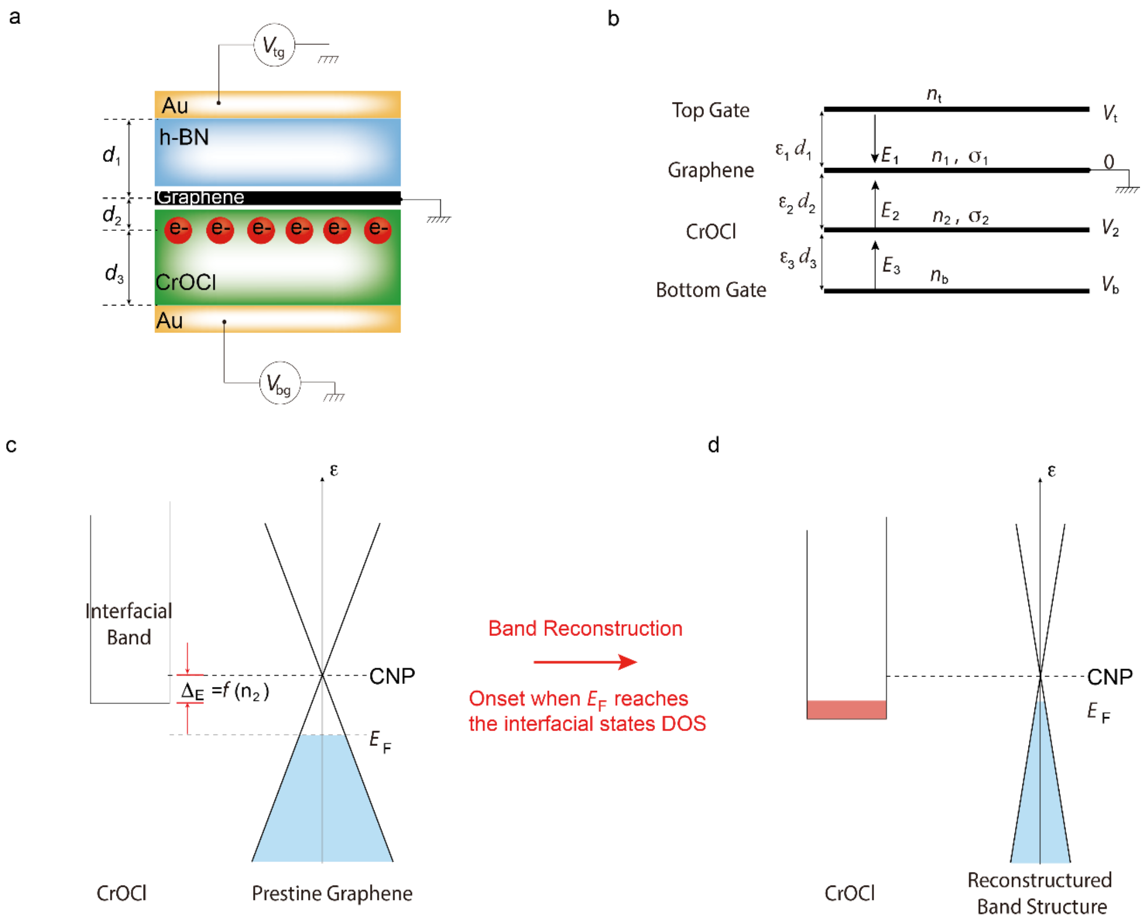

Supplementary Figure 25. Electro-static model of the h-BN/graphene/CrOCl system. (a) The configuration of our studied device, with the assumption of interfacial charge states layer. (b) Simplified model of (a). (c)-(d) Fermi level matching between the interfacial states and graphene, with a reconstruction of Dirac cone from (c) to (d). The Fermi velocity of (d) is estimated to be 6 times higher than that in (c). Another version of such band diagram is given in Fig. 4 in the main text.

When the graphene band is filled with charge carriers, its E_F increases. At the point when E_F matches the lowest value of the interfacial states, electrons start to fill into the DOS of interfacial states (Supplementary Figure 25d). We will argue in the coming parts that to observe the experimentally observed phenomena, there is an enhancement

of Fermi velocity v_F together with an enlargement of Δ_E.

In the coming calculations, DOS for the interfacial states and graphene are written as,

$$\rho_{\mathrm{CrOCl}}(\epsilon) = \rho \qquad (1)$$

$$\rho_{\mathrm{MLG}}(\epsilon) = \frac{4\pi}{\hbar^2 v_F^2}|\epsilon| \qquad (2)$$

We noticed that, experimentally, d_1/ϵ_1 and d_3/ϵ_3 can be almost the same, and both d_1 and d_3 are way larger than d_2. For simplicity, we define

$$d_2 = l \qquad (3)$$

$$d_1/\epsilon_1 = d_3/\epsilon_3 = \alpha l \qquad (4)$$

Here, α is a large dimensionless coefficient.
Electron charge is written as $-e$, with $e=1.6\times10^{-19}$ C.
Each electric field can be written as

$$E_1 = \frac{V_t}{d_1} \qquad (5)$$

$$E_2 = \frac{V_2}{d_2} \qquad (6)$$

$$E_3 = \frac{V_b - V_2}{d_3} \qquad (7)$$

According to the Gauss's law

$$\sigma_1 = -(\epsilon_1 E_1 + \epsilon_2 E_2)\epsilon_0 = -\frac{(V_t + \alpha V_2)\epsilon_0}{\alpha l} \qquad (8)$$

$$\sigma_2 = (\epsilon_2 E_2 - \epsilon_3 E_3)\epsilon_0 = \frac{\left((\alpha+1)V_2 - V_b\right)\epsilon_0}{\alpha l} \qquad (9)$$

and

$$\sigma_i = -n_i e, i = 1,2 \qquad (10)$$

One obtains the following

$$n_1 = \frac{(V_t + \alpha V_2)\epsilon_0}{\alpha l e} \qquad (11)$$

$$n_2 = \frac{(-(\alpha+1)V_2 + V_b)\epsilon_0}{\alpha l e} \qquad (12)$$

because the chemical potentials of the interfacial states and graphene are equal, one gets

$$n_2 = 0 \qquad (13)$$

or

$$\frac{n_2}{\rho} = \Delta E + eV_2 + \mathrm{sgn}(n_1)\sqrt{\frac{\hbar^2 v_F^2}{2\pi}|n_1|} \qquad (14)$$

We then have two different situations, discussed below:

Phase-(i) $\mathbf{n_2} = \mathbf{0}$

According to eq. (2)

$$V_2 = \frac{V_b}{\alpha+1} \tag{15}$$

Put the above relation into eq. (1), one obtains:

$$n_1 = \frac{\epsilon_0}{\alpha l e}(V_t + \frac{\alpha}{\alpha+1}V_b) \tag{16}$$

Phase-(ii) $\mathbf{n_2} > 0$

According to eq. (14)

$$V_2 = V_2(n_1, n_2) \tag{17}$$

And according to (11), (12)

$$V_b = V_b(n_1, n_2) \tag{18}$$

$$V_t = V_t(n_1, n_2) \tag{19}$$

For different values of n_1, increasing of n2 from 0, will yield the iso-doping (at which *n*= constant) lines in the phase of (ii). And eq. (16) can define the iso-doping lines of graphene in the phase of (i).

The parameters used in our calculations are listed below (with S.I. units omitted):

$\alpha = 50$

$l = 2 \times 10^{-10}$

$\epsilon_0 = 8.85 \times 10^{-12}$

$e = 1.602 \times 10^{-19}$

$\rho = 10^{20}/e$

$\Delta E = 0.19e$

$v_F = 1.5 \times 10^6$

$\hbar = 1.05 \times 10^{-34}$

$\pi = 3.1415926$

We will then obtain the iso-doping lines of the graphene/CrOCl system based on the above modellings. It is seen that the phases (i) and (ii) are separated by a green dashed line, as shown in Supplementary Figure 26. However, the iso-doping lines in phase (ii) are straight lines, which do not agree with experimental measurements.

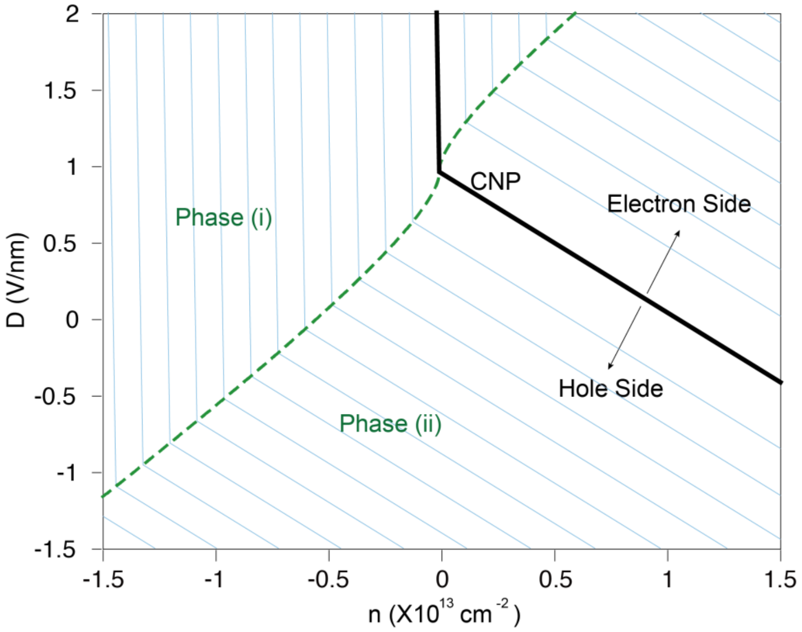

Supplementary Figure 26. Iso-doping lines of the graphene/CrOCl system, with renormalization of neither v_F nor $\Delta E(n_2)$. Difference of carrier density between each iso-doping line (solid light blue lines) is set to be 1.2×10^{12} cm^{-2}.

The DOS of MLG and the interfacial states are $\rho_1(\epsilon)$ and $\rho_2(\epsilon)$ throughout the doping process, then the number of charges can be written as

$$\int_0^x \rho_i(\epsilon)d\epsilon = n_i \tag{20}$$

which defines the relation between n_i and x:

$$x = f_i(n_i), i = 1,2 \tag{21}$$

Clearly, $f_i(n_i)$ has to be a monotonously increasing function.

We now discuss the characteristics of iso-doping lines in MLG, when $n_2 > 0$ (Phase-ii).

In this scenario, eq. (14) turns into:

$$f_2(n_2) = \Delta E + eV_2 + f_1(n_1) \tag{22}$$

In the mean time, we can re-write eq. (11) & (12) as:

$$V_t = \frac{\alpha le}{\epsilon_0} n_1 - \alpha V_2 \tag{23}$$

$$V_b = \frac{\alpha le}{\epsilon_0} n_2 + (\alpha + 1)V_2 \tag{24}$$

Starting from $n_2 = 0$, when increasing n_2, the iso-doping lines of MLG will tend to go straight downwards to lower *D* and higher *n*.

According to Eq.(11)-(13), when at constant $\mathrm{n_1}$, increasing $\mathrm{n_2}$ will lead to an increase of$\mathrm{V_2}$, thus the increasing of $\mathrm{V_b}$, and the decreasing of $\mathrm{V_t}$. However, this does not agree with experimental observations, which show that when increasing $\mathrm{n_2}$, $\mathrm{V_t}$ and $\mathrm{V_b}$ increases simultaneously.

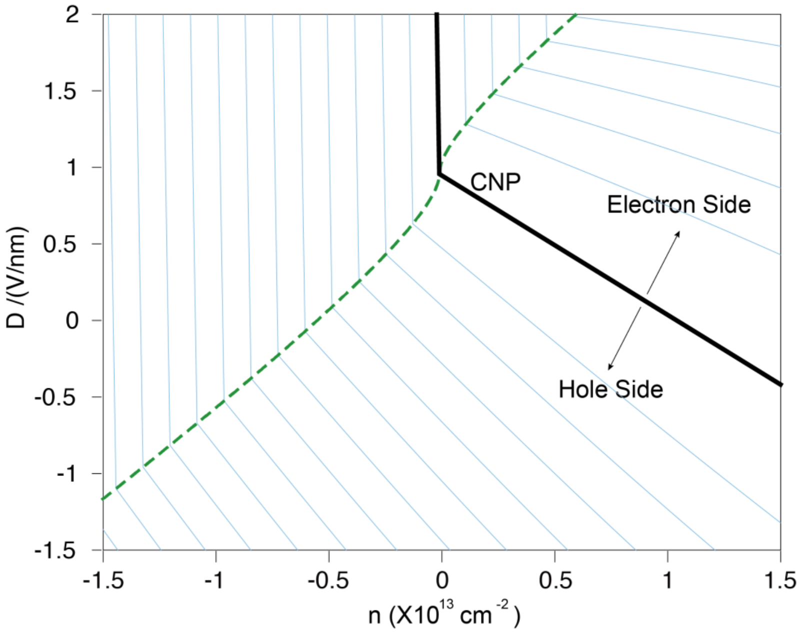

Supplementary Figure 27. Iso-doping lines of the graphene/CrOCl system, with only renormalization of v_F as shown in Eq. 25. The plot gains a distance-increasing feature in the iso-doping lines upon increasing n_{tot}, in the Phase (ii). Difference of carrier density between each iso-doping line (solid light blue lines) is set to be 1.2×10^{12} cm^{-2}.

One of the possibilities is that the bandwidth of the interfacial states gets expanded, and thus $\mathrm{V_2}$ can decrease with increasing $\mathrm{n_2}$. According our experimental data, we have good reasons to assume that the Fermi velocity of graphene v_F is increasing with increasing the $\mathrm{n_2}$, and saturates at a maximum value. We take a simple asymptotic equation for the simplified model, as:

$$v_F = 1.5E6 * \left(1 + 5 * \frac{3E{-}18 * n_2}{\sqrt{(3E{-}18 * n_2)^2 + 1}}\right) \qquad (25)$$

It turns out that except for the CNP, other iso-doping lines in the Phase (ii) start to have a distance-increasing effect upon increasing n_{tot}. Clearly, the CPN are charge neutral and not affected by the v_F.

Notice that the above result depicts that the v_F is enlarged by a factor of ~6 when entering from phase-(i) into phase-(ii). Such v_F (~10^7 m/s) is a record value in all condensed matter systems known thus far, and it is a direct consequence of band

structure reconstruction of the Dirac dispersion in graphene placed on an electron layer in the CrOCl lattice. Why this can happen and how it can be tuned will be further explained by investigations such as DFT calculations, or STM studies.

In order to have the bending effect of CNP, we now assume that ΔE also is a asymptotic function of n_2 (we note that by varying n_2 itself as a asymptotic function can also lead to similar results) which may be a consequence of the bandwidth expansion due to electron interactions in the interfacial states. By taking a hypothesis of a simple asymptotic function, one gets:

$$\Delta E(n_2) = \Delta E(0) + 4.5E - 20 * \frac{1E-17*n_2}{\sqrt{(1E-17*n_2)^2+1}} \qquad (26)$$

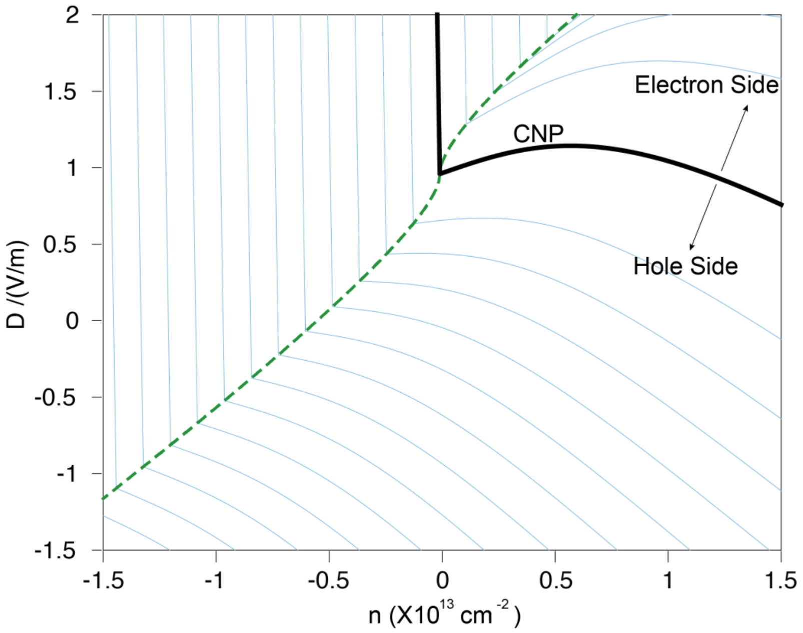

Supplementary Figure 28. Iso-doping lines of the graphene/CrOCl system, with renormalizations of both v_F and $\Delta E(n_2)$, as shown in Eq. 25 and Eq. 26. The up-bending of CNP, as well as distance-increasing in the iso-doping lines upon increasing n_{tot} in the Phase (ii), are well reproduced using our simplified electrostatic model. Difference of carrier density between each iso-doping line (solid light blue lines) is set to be 1.2×10^{12} cm^{-2}.

The updated assumption can be plotted in Supplementary Figure 28. At this stage, our electro-static model can well address the two major experimental findings in our work. One can thus conjecture that the measured exotic quantum Hall phase in the Phase-ii may be a consequence of a subtle interfacial coupling, which may invoke a renormalization of v_F (enhanced by over 6 times in the above model) in graphene, as shown in Supplementary Figure 25. This is in qualitative agreement with the observed QHE at very low magnetic fields.

Supplementary Note 2. DFT calculations with the Hartree-Fock methods

Section 1. DFT model of graphene on monolayer CrOCl

We first investigated by modelling the system into a simplified monolayer CrOCl/graphene structure (see Methods in the main text), whose band structure is illustrated in Supplementary Figure 29. A Dirac dispersion is seen with the Fermi level sitting at the Dirac point between Γ and X points. The band gap of CrOCl is also examined at high electric field, as shown in Supplementary Figure 30.

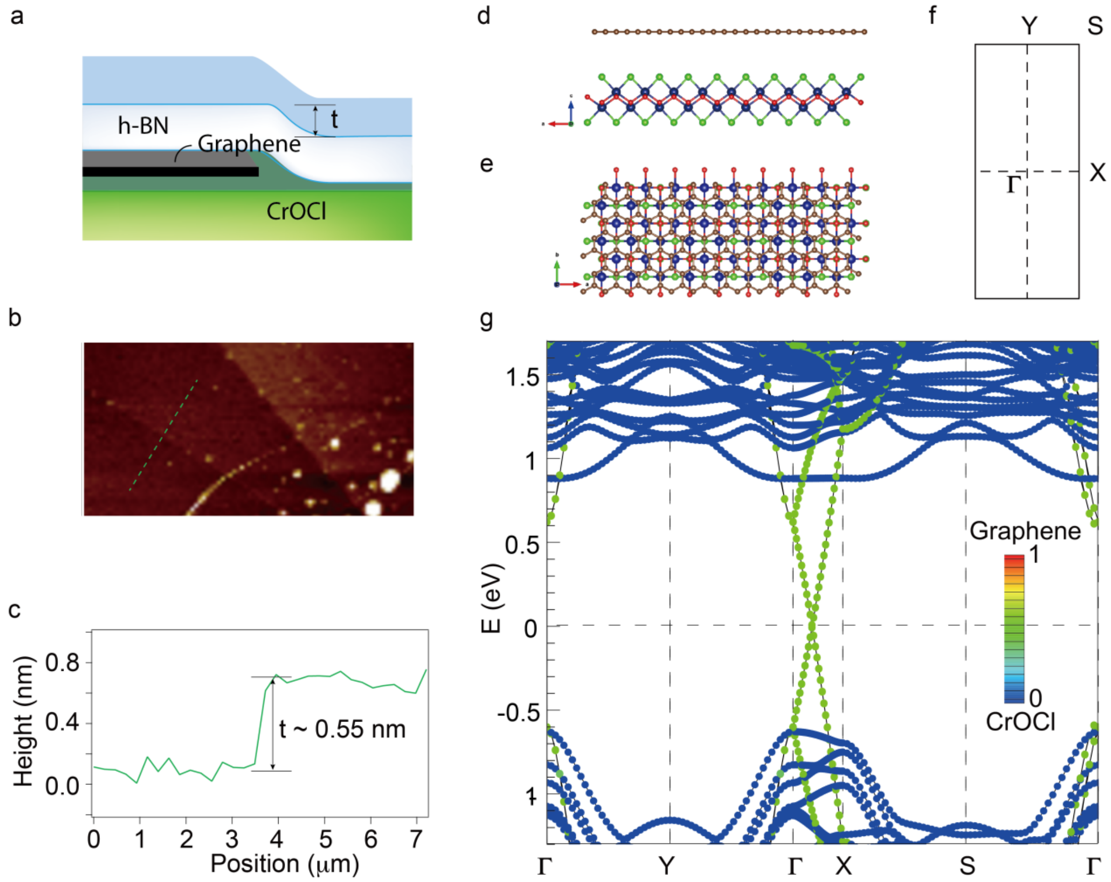

Supplementary Figure 29. Band structure of CrOCl/graphene heterostructure. (a) and (b) are schematic picture and AFM image of the h-BN/graphene/CrOCl hybrid. (c) Height profile along green dashed line in (b). It is seen that around 5 Å distance is estimated from the AFM measurements, and is in agreement with the relaxed result in DFT calculations. (d)-(e) side and top views of the unit cell described in the Methods section in the main text, with a Brillouin zone illustrated in (f). (g) shows the electronic band structure using DFT calculations.

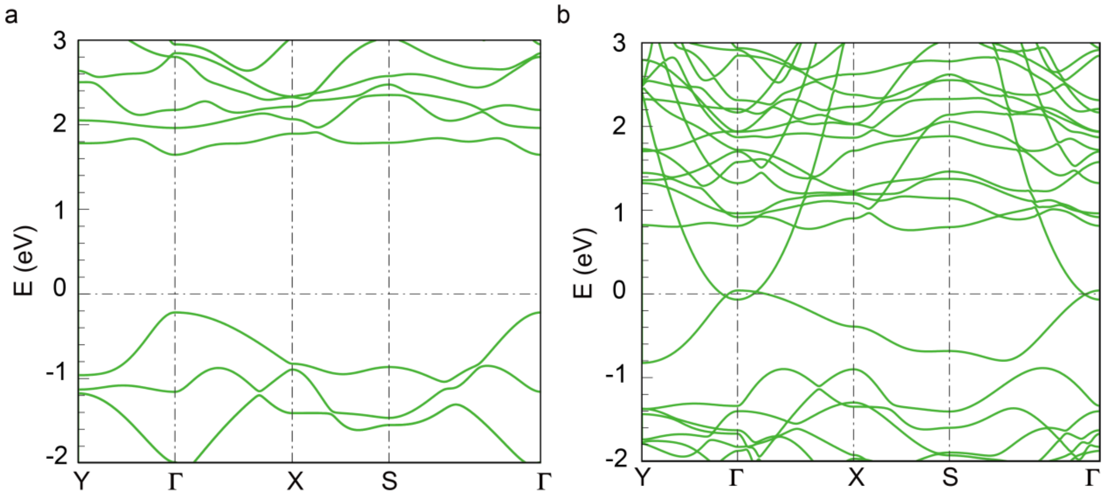

Supplementary Figure 30. **Gap closing of CrOCl under electrical fields**. Band structure of monolayered CrOCl at vertical electrical fields of (a) 0 V/nm, and (b) 5.5 V/nm, respectively. It is seen in (b) that the band gap of CrOCl closes at 5.5 V/nm, but without any topological gap from band inversion.

Section 2. DFT model of graphene on bilayer CrOCl

In the previous DFT modeling in Section 1, we used a single layer CrOCl and the conduction band around the Fermi level can be seen at an electric field of ~5.5 V/nm (Supplementary Figure 30b). However, it was not clear in the sense of addressing the interfacial band since the surface band cannot be distinguished from different Cr atoms in different layers along the direction of applied electrical field. We now considered bilayer CrOCl which can better address the physical process as there is a well-defined *z*-direction gradient of electrostatic potential for Cr atoms in different layers (3*d*-orbitals of Cr contributes mainly near the Fermi level).

In order to make this section more pedagogic, we will introduce our main new arguments in two points:

(A) The interfacial band exhibits no contribution to transport.

(B) There is enhancement of Fermi velocity in graphene, due to the interaction from the interfacial band.

First, we go through our previous simplified electrostatic model in Supplementary Note 1: the interfacial band emerged in CrOCl under finite vertical electric field has to be a band that does not contribute to transport (otherwise it will shut the graphene and change the Dirac-like transport and the measured LLs). This assumption was used in our simplified model together with the other assumption of an enhanced Fermi velocity, in order to self-consistently explain the key experimental features, as discussed in the Supplementary Note 1.

It is known that, in some specific cases, 2D electron gases can be crystallized exhibiting an insulating phase that does not participate in transport. Two typical examples are charge density wave (CDW) and Wigner crystal, which are strongly localized electrons with long range order. Indeed, experimental reports have suggested that localized CDW states can be found in other systems such as 1T-TaS_2 interfaced graphene.[3] We thus expect similar long wavelength electronic crystal may be possible in our system, and below we will show the proof.

To elucidate (A), we carried out the first principles calculations based on density functional theory (DFT) using Vienna *Ab initio* Simulation Package (VASP) with projector augmented wave method. The generalized gradient approximation (GGA) by Perdew, Burke, and Ernzerhof is taken as the exchange–correlation potential. Since Cr is a transition metal element with localized 3*d* orbitals, the on-site Hubbard U = 2.7 eV parameter is used in the calculations.

[3] Altvater, M. A. *et al.*, "Revealing the Charge Density Wave Proximity Effect in Graphene on 1T-TaS_2", arXiv: 2201.09195 (2022).

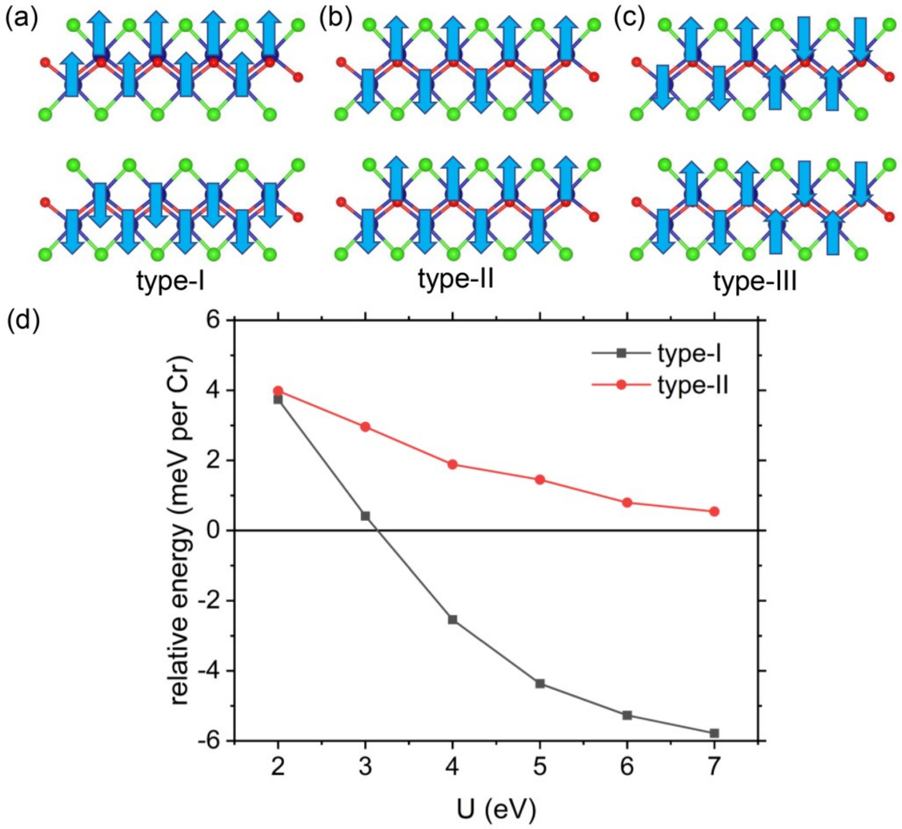

Supplementary Figure 31. We present the three possible types of magnetic orders in the bilayer CrOCl in the figure (a),(b),and (c), respectively. The total energies of type-I and type-II magnetic states as a function of on-site Hubbard *U* value are shown in the (d). Here the total energies of type-III magnetic state at different *U* values are set to zeros.

To study the system under electric fields, we have to consider at least a bilayer CrOCl (see Supplementary Figure 31) because monolayer is indifferent to vertical electric field. We set an interlayer antiferromagnetic (AFM) order and an intralayer ferromagnetic (FM) order to the bilayer CrOCl system, which turns out to be one of the competing magnetic ground states for bilayer CrOCl. To be specific, we have calculated the energies of three magnetic configurations: (I) an inter-layer AFM and intralayer FM state as shown in Supplementary Figure 31a; (II) an intralayer AFM and interlayer FM state as shown in Supplementary Figure 31b; and (III) a 1× 4× 1 intralayer AFM and interlayer FM state that enlarges the primitive cell by four times along the in-plane *b*-axis. Previous first principles calculations show that the type (III) intralayer AFM state is the bulk ground state with the Hubbard *U*= 3.2 eV calculated from constrained random phase approximation.[4] However, our calculations indicate that the type (I)

[4] S. W. Jang, D. H. Kiem, J. Lee, Y.-G. Kang, H. Yoon, and M. J. Han, Phys. Rev. Materials 5, 034409 (2021).

interlayer AFM state, which has never been considered in previous studies, is a also candidate ground state that competes with the type (III) intralayer AFM state. As shown in Supplementary Figure 31d, as the Hubbard U value is increased, the energy of the type (I) becomes lower than that of the type (III) state (set to zero) when U $\gtrsim$ 3 eV. This indicates that the type (I) interlayer AFM state is likely to be the magnetic ground state, especially for the CrOCl substrate surrounded by the complex dielectric environment in our device.

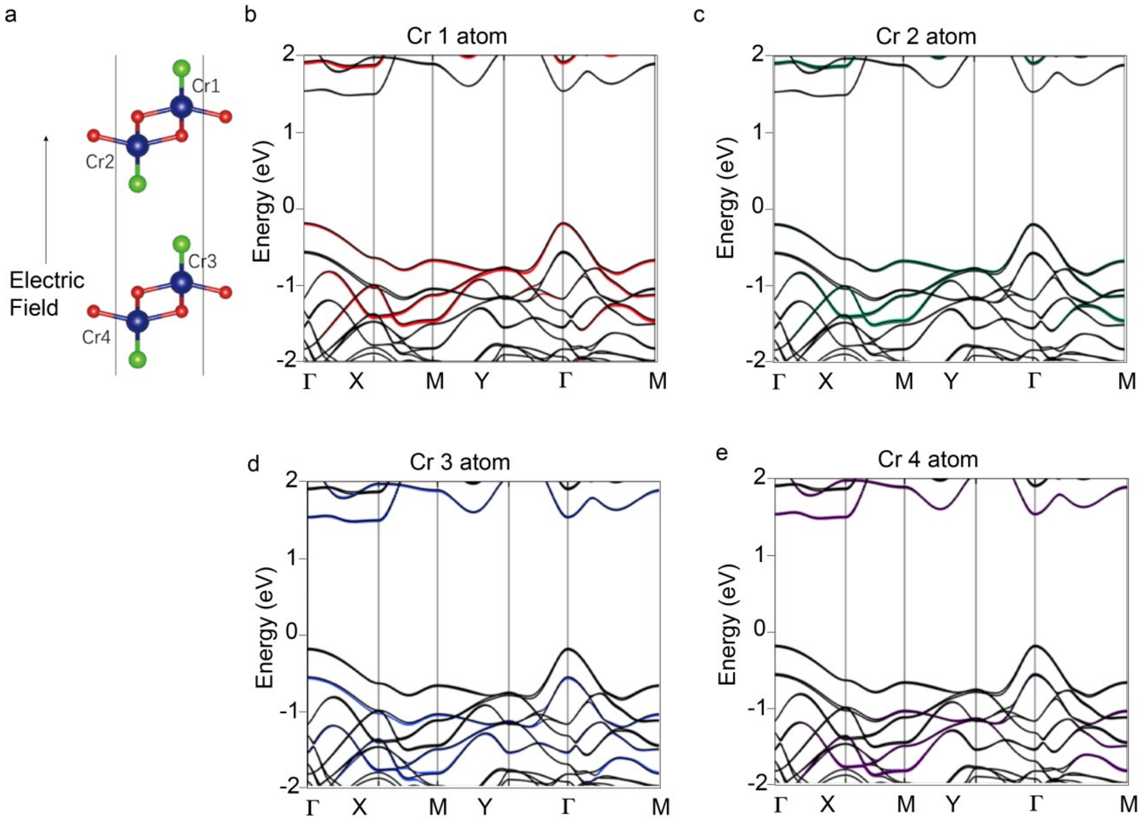

Supplementary Figure 32. The energy bands of bilayer CrOCl projected onto each Cr atom at electrical field of *E*=0.08 V/nm. (a) Side view of the atomic structure of the bilayer CrOCl, electric fields are applied along *c*-axis for calculating the resulted band structure. (b)-(e) Projected bands of each Cr atoms (red, green, blue and purple) in the total bands (black). Type-II lattice with a vertical electric field of 0.08 V/nm was included in the calculation.

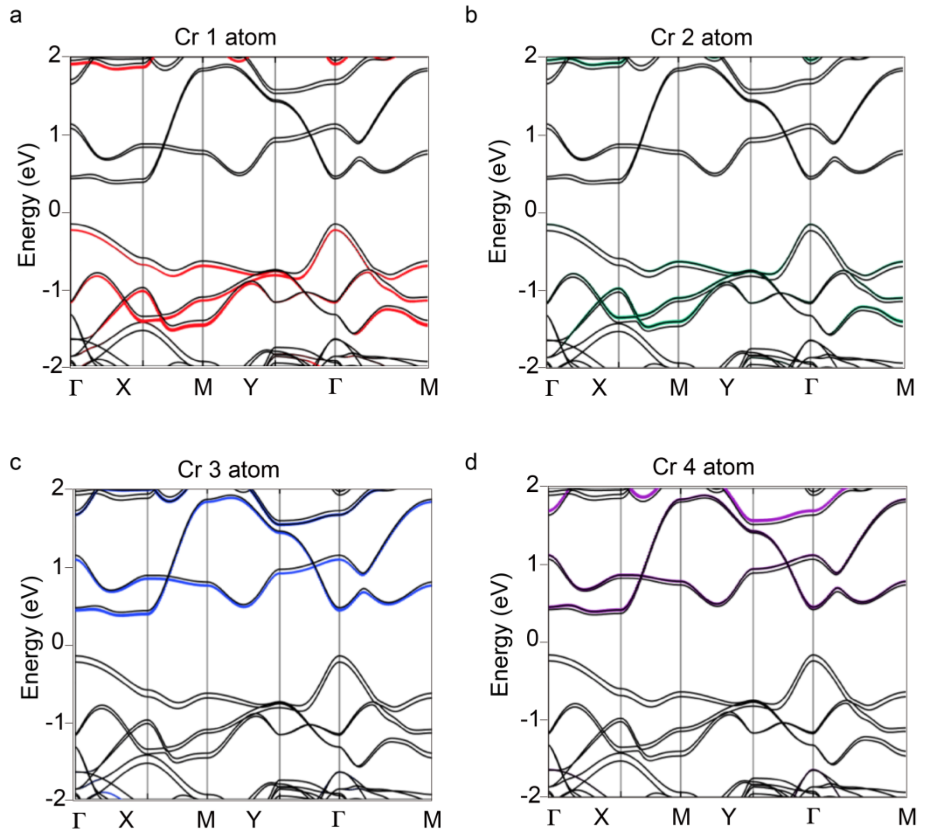

Supplementary Figure 33. The energy bands of bilayer CrOCl projected onto each Cr atom at electric field of *D*=0.32 V/nm. (a) -(d) Projected bands of each Cr atoms (red, green, blue and purple) in the total bands (black). A vertical electric field of 0.32 V/nm was applied in the calculation within the type-II lattice. It is seen that the conduction bands (contributed by Cr3-4 atoms, *i.e.*, the top layer) are pull down to lower energy with respect to those shown in Supplementary Figure 32.

Taking type-II magnetic configuration for example, to investigate the electric field's influences on the electronic structures, we consider the screened electric fields of 0.08 V/nm and 0.32 V/nm, and the corresponding DFT band structures of the bilayer CrOCl under two of the electric fields are shown in Supplementary Figure 32-33. The thickness of the vacuum region is set as 40 Å to avoid any artificial interactions. It is seen that by applying a positive electric field, an interfacial band from top layer of Cr atoms is available at much lower energy, which are mostly contributed by the bottom layer Cr *3d* orbitals (Supplementary Figure 33). In Supplementary Figure 34 we plot together the band of monolayer graphene and bilayer CrOCl in a commensurate super-cell. It is clear in Supplementary Figure 34b that at certain electric field (*D*=0.32V/nm), the interfacial band from the top layer of CrOCl (mainly from the top Cr atoms, as more details can be found in the following) starts to overlap with the Dirac point of graphene,

this is the event that charge transfer (i.e., filling of electrons in the interfacial band) from graphene to the interfacial band is triggered. As will be discussed later, as the conduction band of the CrOCl has a large effective mass (see Supplementary Table 1), slight charge transfer from graphene to the interfacial band at the surface of CrOCl may result in a long-wavelength charge ordered state through Wigner crystallization scenario.

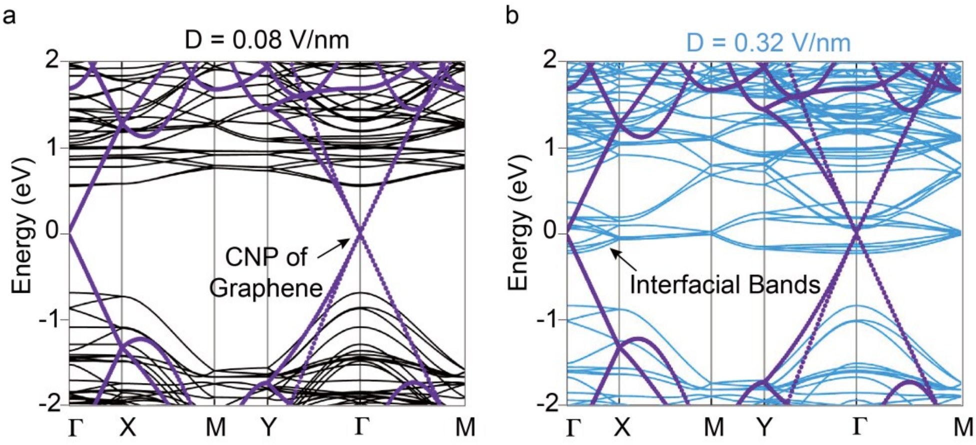

Supplementary Figure 34. The energy bands of bilayer CrOCl/graphene heterostructure under electric field of 0.08 V/nm (a), and 0.32 V/nm (b). The relative energies of graphene monolayer are marked by purple dots.

Then the CrOCl-graphene system is modeled as a long-wavelength charge order that is coupled with the Dirac fermions of graphene. The charge ordered state in the top surface of CrOCl substrate is supposed to be gapped as otherwise it would contribute to the transport data, which is not observed in experiments. Thus, if we are only interested in the low-energy physics, it is legitimate to integrate out the charge degrees of freedom on the CrOCl side, and assume that the charge order only exerts a Coulomb potential to the Dirac fermions in the graphene monolayer.[5] Then we can construct an effective Hamiltonian for Dirac fermions in a given valley $\tau=\pm$ of graphene that are coupled with an effective background superlattice potential (arising from the Coulomb potentials of the charge order in the CrOCl substrate):

$$H_{\text{eff}}^{\tau} = \hbar v_{\text{F}} \boldsymbol{k} \cdot \sigma^{\tau} + U_{\text{d}}(\boldsymbol{r}) \qquad \text{(S1)}$$

where σ^{τ} are the Pauli matrices ($\tau\sigma_x$, σ_y) and $U_{\text{d}}(\boldsymbol{r})$ is the background superlattice potential with the period $U_{\text{d}}(\boldsymbol{r})=U_{\text{d}}(\boldsymbol{r}+\boldsymbol{R}_s)$, whose Fourier transform reads

[5] Lu X., *et al.*, "Synergistic interplay between Dirac fermion and long-wavelength order parameters in graphene-insulator heterostructures", arXiv:2206.05659 (2022).

$U(Q)=\frac{e^2 e^{-Qd_2}}{\varepsilon_0\varepsilon_d\Omega_0 Q}$, where the superlattice vector $\boldsymbol{R_s}$ is expected to bemuch larger than any atomic lattice constant due to the low carrier density, **Q** denotes a reciprocal vector of the superlattice, Ω_0 denotes the area of the primitive supercell, ε_0 denotes vacuum permittivity, ε_d is a background dielectric constant characterizing the screening of the Coulomb potential. The superlattice constant is set to be around L_s=50Å derived from the nominal doping ~10^{12}-10^{13} cm^{-2}. Since L_s is much larger than the lattice constant of graphene, we could thus safely omit the intervalley coupling and model graphene as two separate continua of Dirac fermions from two valleys. As a result, the underlying superlattice would fold Dirac cones into its small Brillouin zone forming subbands. Technically, the spacing between Cr atoms and graphene is found to be d_2=7 Å from a DFT lattice relaxation study. We assume that the coupling is only via the long-ranged Coulomb interactions, *i.e.*, neglecting the coupling from orbital overlaps such as interlayer hoppings, which is screened by dielectric constants ε_d = 3-5. A detailed derivation of the continuum model (Eq. S1) and the superlattice Coulomb potential can be found in Ref. [5].

As briefly discussed above, such long-wavelength charge order arises from the interface charge transfer between CrOCl and graphene, such that the surface conduction band of CrOCl becomes slightly doped under vertical displacement fields, leading to some Wigner-crystal-like instabilities. This is taking effect because electrons in the top surface states (transferred from graphene) have a very low density (10^{12} cm^{-2}, as deduced from the nominal doping, and the associated dimensionless Wigner-Seitz radius r_s = 47 with an effective mass m^* = 2:24 m_e and a dielectric constant ε_r = 5) so that interactions would prevail over the kinetic energy.

Supplementary Table 1. Effective masses and estimated Wigner-Seitz radii in bilayer CrOCl with different types of magnetic ordering under slight doping concentration of ~ 10^{12} cm^{-2}.

Lattice Type	I	II	III
**Effective Mass (m_e)**	1.308	2.245	6.772
**Wigner-Seitz radii (ε_r = 3)**	65.7	112.7	399.9
**Wigner-Seitz radii (ε_r = 5)**	39.4	67.6	203.9

It is worth mentioning that, in our DFT calculations indicate that the distance between graphene layer and the top surface of CrOCl is 0.7nm, while the 3*d* orbitals of Cr (which

contributes to the interfacial states and the top surface of CrOCl) are well localized in real space whose spatial spread ≤0.1nm. It's exactly because of the negligible wavefunction overlap between the CrOCl interfacial state and the Dirac state of graphene, the CrOCl substrate has negligible magnetic exchange effect on the graphene layer. For the same reason, the localized charges in the long-wavelength ordered state at the surface CrOCl does not contribute to any transport phenomena. Instead, as the localized long-wavelength charge order in the CrOCl still interacts with the Dirac electrons via long-range Coulomb interactions, which decays as 1/r (r is the distance). Moreover, our DFT results suggest that different magnetic ground state does not affect the charge filling in the surface interfacial band in CrOCl, as the Wigner crystal is mainly determined by the interfacial charge transfer tuned by electric fields. Therefore, we argue that the dominant interaction between the interfacial state of CrOCl and Dirac electrons of graphene is the long-range Coulomb interactions, and the exponentially decaying magnetic exchange interaction can be neglected. Although a number of works have focused on the magnetic phase transition on CrOCl itself.[6,7,8,9,10,11,12]

Section 3. Hartree-Fock calculations.

To address (B) mentioned in Section 2, we have performed the interacting band structure calculation of graphene by using the continuum-model-based unrestricted Hartree-Fock method, which is presented in a separate theoretical work Ref. [5], and here we make a brief summary of the key results.

We investigate *e-e* interaction effects in the graphene-CrOCl heterostructure using the Hartree-Fock approximations, where the non-interacting low-energy physics is described by the continuum Hamiltonian of Dirac fermions coupled to a long-wavelength superlattice potential (Eq. (S1)). Then the e-e interaction between the

---

[6] Christensen, A. N., Johansson, T. & Qu ezel, S. Preparation and magnetic properties of CrOCl. Acta. Shem. Scand. 28, 1171 (1975).

[7] Zhang, T. *et al.* Magnetism and optical anisotropy in van der Waals antiferromagnetic insulator CrOCl. ACSNano 13, 11353-11362 (2019).

[8] Gu, P. *et al.* Magnetic phase transitions and magnetoelastic coupling in a two-dimensional stripy antiferromagnet. Nano letters 22, 1233 (2022).

[9] Miao, N. *et al.*, "2D Intrinsic Ferromagnets from van der Waals Antiferromagnets", J. Am. Chem. Soc. 140, 2417–2420 (2018).

[10] Qing, X. *et al.*, "Magnetism and spin exchange coupling in strained monolayer CrOCl", Phys. Chem. Chem. Phys., 22, 17255-17262 (2020).

[11] Nair, A. K., *et al.*, "Bi-stimuli assisted engineering and control of magnetic phase in monolayer CrOCl", Phys. Chem. Chem. Phys. 22, 12806 (2020).

[12] Seung, W. J. *et al.*, "Hund's physics and the magnetic ground state of CrOX (X = Cl, Br), arXiv:2102.10729 (2021).

low-energy Dirac electrons is considered, which is set as in a double-gate screened form, and is expressed in Fourier space as $V(\boldsymbol{q}) = \dfrac{e^2 \tanh(qd_s)}{2\varepsilon_0 \varepsilon_d \Omega_0 q}$, with a background dielectric constant ε_d = 3-5 and the thickness of the insulating substrate d_s=400Å. The Coulomb interactions are then expressed in the subband eigenfunction basis. As interaction effects are most prominent around the CNP, we project the Coulomb interactions onto only a low-energy window including five valence and three conduction subbands that are closest to the Dirac point for each valley and spin. We use a mesh of 18 × 18 ***k***-points to sample the mini Brillouin zone of the superlattice. To incorporate the influences of Coulomb interactions from the high-energy remote bands, we rescale the Fermi velocity within the low-energy window of the effective Hamiltonian using the formula derived from the renormalization group approach.[13] We keep other parameters of the non-interacting effective Hamiltonian unchanged since their renormalization-group correction is of higher order, thus can be neglected.[5] Feeding with the initial conditions in the form of order parameters, we can self-consistently obtain the gap at the CNP and the single-particle excitation spectrum.

Our Hartree-Fock calculations show that a gap (on the order of a few meV) can be opened at the CNP in the SIC-phase, and the Fermi velocity at a slight filling around 10^{11} cm^{-2} can be magnified by a factor of 2,[5] both are qualitatively consistent with experiments. As a gap of a few meV can be extracted by thermal activation in Supplementary Figure 35, and the Fermi velocity in Phase-ii is indeed estimated to be a few times higher than that of Phase-I according to the SdH analysis in Supplementary Figure 36.

To the best of our knowledge, in contrast to our system, no gap has been experimentally observed in suspended graphene close to the CNP because the increased Fermi velocity near the CNP suppresses the emergence of any gapped states.[14] This suggests that the charge-ordered superlattice from CrOCl substrate may be crucial to the gap opening. Intuitively, the superlattice potential from the long-wavelength charge order in CrOCl confines Dirac fermions, thus reduces the Fermi velocity already without interactions in Ref. [5]. A gap could nevertheless be opened even though Fermi velocity would still be augmented by interactions.

[13]J. Gonzalez, F. Guinea, and V. M. A. H., Nuclear Physics B 424, 595 (1994), ISSN 0550-3213.
[14] D. C. Elias, R. Gorbachev, A. Mayorov, S. Morozov, A. Zhukov, P. Blake, L. Ponomarenko, I. Grigorieva, K. Novoselov, F. Guinea, et al., Nature Physics 7, 701 (2011).

16. Thermal gap extracted from the CNP at the SIC-phase

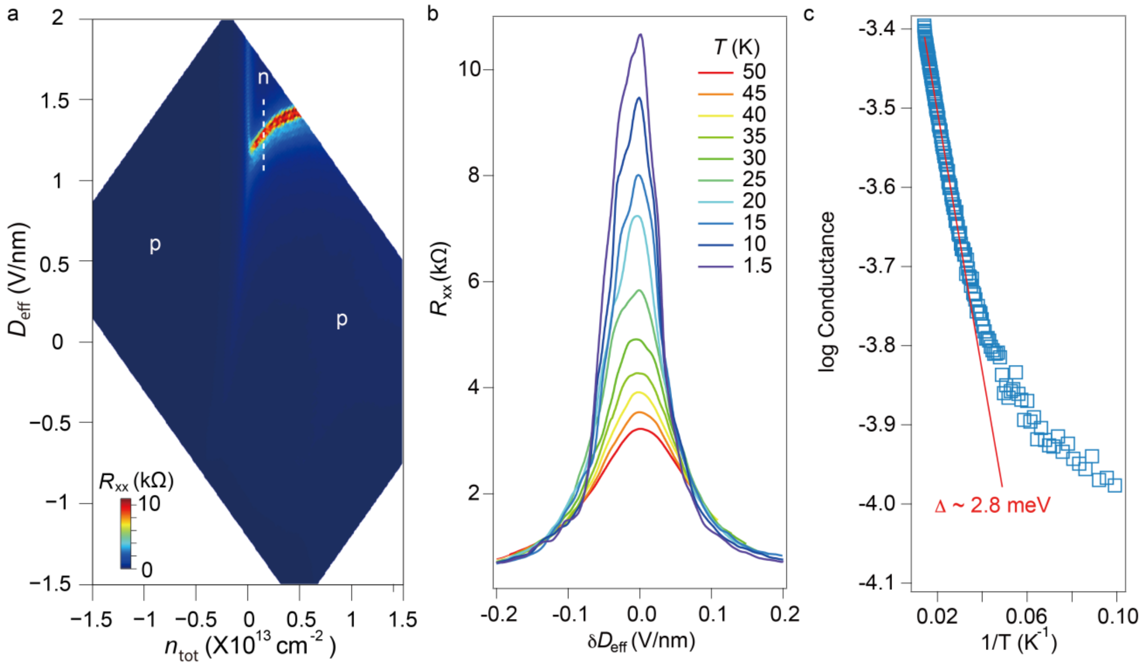

Supplementary Figure 35. Gapped state at the CNP in graphene in Phase-ii. (a) Channel resistance R_{xx} measured in the space of D_{eff}-n_{tot} at T = 1.5 K and B = 0 T. (b) Line profiles of the R_{xx}-δD_{eff} (δD_{eff} is renormalized with respect to the D_{eff} at maximum R_{xx}), measured at different temperatures. (c) Log scale of the minimum conductance σ, *i.e.*, log(1/R_{xx}), plotted against 1/T. The thermal activation gap is estimated to be around 2.8 meV using the formula $\sigma \sim \exp(\Delta/2k_BT)$, where k_B is the Boltzmann constant, T is temperature.

17. Shubnikov-de Haas (SdH) analysis of the h-BN/graphene/CrOCl heterostructures

We measured typical SdH curves in Device-S16, S17, and S40.

Taking Device-S40 for example, we measured SdH at δD_{eff}=-0.4 V/nm, and n_{tot} was set to be -2.5×10^{12} cm^{-2} and $+2.5\times10^{12}$ cm^{-2} for Phase-i and Phase-ii, respectively. It is seen that the amplitudes of quantum oscillations in Phase-i (Supplementary Figure 36a) disappear at 80 K, while in Phase-ii (Supplementary Figure 36b) the oscillation strikingly persists up to 120 K with considerably large amplitude. It is regrettable that at even higher temperatures the device started to have gate leakage, therefore we monitored the SdH at maximum temperature of 120 K in these tested devices.

We then performed the Lifshitz-Kosevich (L-K) fitting of the samples to estimate the cyclotron masses. The temperature dependence of the resistivity oscillations amplitude $A=\Delta\rho_{xx}(T)$ is given by the L-K formula $\Delta\rho_{xx}(T)\propto(2\pi^2k_BTm^*/\hbar eB)/\sinh(2\pi^2k_BTm^*/\hbar eB)$, where k_B is the Boltzmann constant and m^* is the cyclotron mass, e is the elementary charge and B is the magnetic field.[15] The formula can be approximated into a linear relation of ln(A/T) vs. T, with the slope being a measure of the cyclotron mass. In Supplementary Figure 36c, we show two typical L-K fit for the data in Phase-i and Phase-ii in Device-S40, at 2.8 T and 1.7 T, respectively. The fitted cyclotron mass in Phase-ii is about 5 times smaller than that in Phase-i, which is in agreement with our theoretical predictions in Ref. [5]. A summarization of cyclotron mass in unit of electron mass m_e extracted using L-K fitting in different n_{tot} and D_{eff} in several typical samples is given in Fig. Supplementary Figure 36d.

[15] Shoenberg, D. Magnetic Oscillations in Metals (Cambridge University Press, Cambridge, 1984).

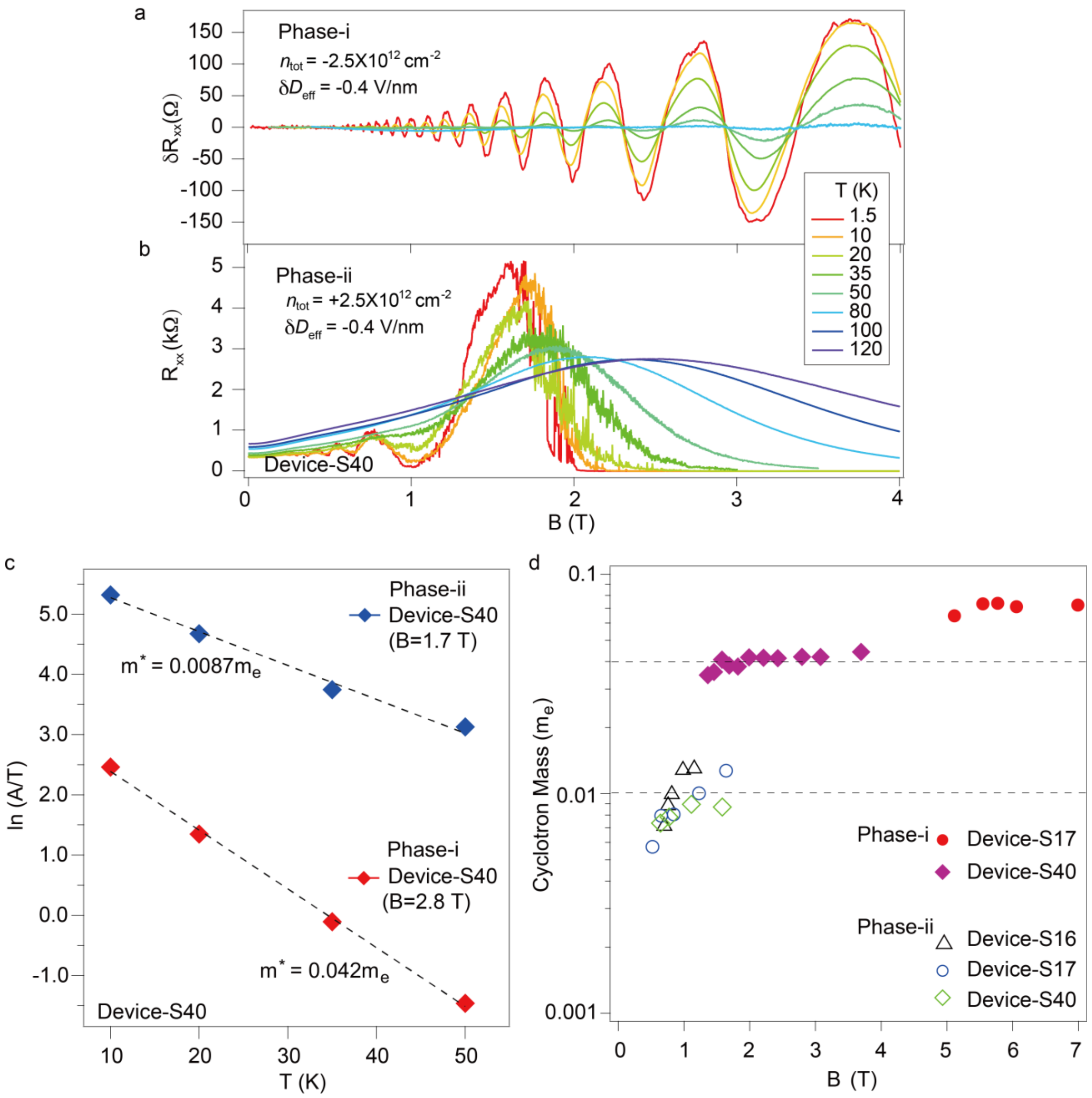

Supplementary Figure 36. Shubnikov-de Haas (SdH) analysis of typical dual-gated CrOCl/graphene/h-BN samples. (a)-(b) The Shubnikov-de Haas (SdH) oscillations from various temperatures at dopings of n_{tot} $\pm 2.5\times10^{12}$ cm^{-2} and D_{eff}=-0.4 V/nm in Device-S40. (c) Lifshitz-Kosevich fit in Phase-i and Phase-ii in Device-S40, at 2.8 T and 1.7 T, respectively. (d) Cyclotron masses obtained in several samples. It is seen that the m^* in Phase-I in our system is in agreement with the "ordinary" monolayer graphene reported elsewhere.[16,17,18]

---

[16] Zhang, Y., Tan, Y. W., Stormer, H. L. & Kim, P., Experimental observation of the quantum hall effect and berry's phase in graphene. Nature 438, 201 (2005).
[17] Novoselov, K. S. *et al.* Two-dimensional gas of massless Dirac fermions in graphene. Nature 438, 197 (2005).
[18] Tan, Z. *et al.* Shubnikov-de Haas oscillations of a single layer graphene under dc current bias. Phys. Rev. B 84, 115429 (2011).
